# Amuc_1100 alleviates HFD-induced hepatic lipid accumulation via gut microbiota in zebrafish: insights from the role of intestinal 14-3-3β/α-A

**DOI:** 10.1186/s40104-026-01418-7

**Published:** 2026-06-10

**Authors:** Qianwen Ding, Ruobing Lou, Hui Liang, Yi Wang, Shangshang Tang, Jian Zhang, Yihui Du, Chao Ran, Yalin Yang, Zhen Zhang, Yuanyuan Yao, Chen Wang, Zhen Liu, Oliana Carnevali, Fengli Zhang, Zhigang Zhou

**Affiliations:** 1 China-Norway Joint Lab on Fish Gastrointestinal Microbiota, Institute of Feed Research, Chinese Academy of Agricultural Sciences, Beijing, 100081 People’s Republic of China; 2 National Collection of Livestock and Aquatic Microbes, Institute of Feed Research, Chinese Academy of Agricultural Sciences, Beijing, 100081 People’s Republic of China; 3 Key Laboratory for Feed Biotechnology of the Ministry of Agriculture and Rural Affairs, Institute of Feed Research, Chinese Academy of Agricultural Sciences, Beijing, 100081 People’s Republic of China; 4 National Key Laboratory of Agricultural Microbiology, Biotechnology Research Institute, Chinese Academy of Agricultural Sciences, Beijing, 100081 People’s Republic of China; 5 Hunan Provincial Key Laboratory of Nutrition and Quality Control of Aquatic Animals, Department of Biological and Environmental Engineering, Changsha University, Changsha, 410022 People’s Republic of China; 6 Department of Life and Environmental Sciences, Università Politecnica Delle Marche, Via Brecce Bianche, Ancona, 60131 Italy; 7 Department of Aquaculture, Zhejiang Ocean University, Zhoushan, 316000 People’s Republic of China

**Keywords:** Amuc_1100, Gut microbiota, Hepatic lipid accumulation, Zebrafish, 14-3-3β/α-A

## Abstract

**Background:**

Amuc_1100, the most abundant outer membrane protein of *Akkermansia muciniphila*, alleviates high-fat diet (HFD)-induced hepatic lipid accumulation and modulates gut microbiota in fish; however, its mechanism and mediators remain unknown. Using zebrafish model, this study aims to determine the mechanism by which Amuc_1100 reduces HFD-induced hepatic lipid accumulation through modulation of gut microbiota.

**Methods:**

In main study, 1-month-old zebrafish were fed a low-fat diet (LFD), HFD, or HFD supplemented with 0.01% Amuc_1100 (AM0.01) for 4 weeks. Body weight gain, hepatic lipid accumulation, microbial translocation, and gut microbiota composition were evaluated. In parallel, larvae at 5 d post-fertilization were fed the same diets for 7 d and analyzed by Oil Red O staining. In validation experiments, germ-free (GF) zebrafish received microbiota transplants from donor fish fed HFD or AM0.01. Antibiotics (ABS)-treated zebrafish were fed LFD, HFD, or AM0.01 for 4 weeks. Intestinal protein interacting with Amuc_1100 was identified via pull-down and co-immunoprecipitation, and its role was confirmed using protein-protein interaction (PPI) inhibitor BV02 and gene knockdown. Data were analyzed by Student’s *t*-test or one-way ANOVA.

**Results:**

Compared with HFD group, zebrafish in AM0.01 group showed lower body weight gain, reduced hepatic lipid accumulation, and decreased microbial translocation (*P* < 0.05). AM0.01 feeding increased *Bacillus* abundance while reducing *Acinetobacter*, *Plesiomonas* and *Aeromonas* abundances relative to HFD (*P* < 0.05). GF zebrafish colonized with microbiota from AM0.01-fed donors showed less hepatic lipid accumulation than those receiving microbiota from HFD-fed donors (*P* < 0.05). In contrast, ABS-treated zebrafish showed no significant difference in hepatic triacylglycerol content between HFD and AM0.01 groups (*P* > 0.05). Using pull-down assays with intestinal proteins from LFD-fed zebrafish, we identified 14-3-3β/α-A as an interacting protein of Amuc_1100. When 14-3-3β/α-A PPI was inhibited by BV02, Amuc_1100 failed to alter the HFD-induced gut microbiota profile in 1-month-old zebrafish (*P* > 0.05). Moreover, either BV02 treatment or 14-3-3β/α-A knockdown abolished the protective effect of Amuc_1100 against hepatic lipid accumulation in conventional and GF zebrafish (*P* < 0.05).

**Conclusions:**

Amuc_1100 reduces hepatic lipid accumulation by modulating gut microbiota through intestinal 14-3-3β/α-A, highlighting its potential as a therapeutic target.

**Supplementary Information:**

The online version contains supplementary material available at 10.1186/s40104-026-01418-7.

## Introduction

In recent decades, aquaculture production has expanded rapidly to meet the growing demand for aquatic products, with China contributing approximately 60% of global output [1, 2]. To enhance production efficiency and reduce feed costs, high-fat diets (HFDs) are commonly used in aquaculture. However, this practice often results in an overload of free fatty acids (FFAs), which promotes hepatic lipid accumulation in fish [3–5]. In hybrid grouper, a 15% fat-containing HFD over 8 weeks promoted hepatic lipid droplet accumulation [3]. Yellow catfish fed a 16% fat-containing HFD for 10 weeks developed hepatic lipid metabolism dysfunction and oxidative stress [4], while a 21% fat-containing HFD administered for 60 d profoundly disrupted lipid metabolism and triggered oxidative stress and inflammation [5]. These studies across multiple fish species demonstrate that HFD-induced hepatic steatosis and metabolic disturbances are significant challenges facing the aquaculture industry.

To address the adverse effects of HFD feeding, studies have investigated the potential of probiotics or postbiotics to reduce the occurrence of hepatic lipid accumulation in economically important fish species [6, 7]. Compound probiotics (*Lactobacillus plantarum*:*Saccharomyces cerevisiae*, 9:1) alleviate hepatic lipid accumulation in *Coilia nasus* by enhancing fatty acid oxidation and suppressing fatty acid synthesis [6]. Dietary supplementation with *Lactobacillus rhamnosus* GCC-3 fermentation product reduces hepatic steatosis and inflammation in farmed tilapia (*Oreochromis niloticus*) [7]. Paraprobiotic and postbiotic forms of *Bacillus altitudinis* were shown to reduce liver fat deposition and improve liver health in largemouth bass (*Micropterus salmoides*) fed high-starch diets [8]. Moreover, *Bacillus subtilis* HGCC-1 was found to alleviate hepatic steatosis in golden pompano (*Trachinotus ovatus*) through modulation of gut microbiota and promotion of hepatic lipid utilization [9]. These findings collectively underscore the potential of probiotics and postbiotics in managing HFD-induced hepatic lipid disorders in farmed fish.

*Akkermansia muciniphila* (type strain MucT; ATCC BAA-835), a Gram-negative bacterium characterized by its oxygen-tolerant anaerobic nature, non-motility, non-spore-formation and oval shape, was first isolated in 2004 and classified within the phylum Verrucomicrobia [10, 11]. Owing to its beneficial effects in alleviating metabolic, infectious, and neurological diseases, it is regarded as a promising next-generation probiotic [12–14]. Nevertheless, live *A. muciniphila* can act as a double-edged sword due to its mucin-degrading activity, which disrupts mucus homeostasis and exacerbates pathologies such as food allergic responses, inflammatory bowel disease, and colorectal cancer by promoting type-2 immune responses and facilitating invasion of adherent-invasive microbes [15–17]. Pasteurized forms of the bacterium can promote *Salmonella* infection by inducing the differentiation of intestinal stem cells into microfold cells, which are characterized by a thinner mucus layer [18]. To circumvent these concerns, research has shifted toward identifying specific functional components derived from this bacterium [19]. Amuc_1100, the most abundant outer membrane protein of *A. muciniphila*, has emerged as a key bioactive moiety [19]. Amuc_1100 retains the beneficial properties of *A. muciniphila* while avoiding the risks associated with mucin degradation [19, 20]. This distinction supports the development of Amuc_1100 as a safer alternative to live *A. muciniphila* for therapeutic applications.

In mammalian models, Amuc_1100 has demonstrated activity in improving hepatic lipid metabolism and reducing the occurrence of hepatic inflammation [21, 22]. Plovier et al. first reported that Amuc_1100 treatment can partially recapitulate the metabolic benefits of live *A. muciniphila* in obese and diabetic mice, reducing fat mass development and improving insulin sensitivity [20]. Subsequent studies have confirmed these findings that Amuc_1100 prevents HFD-induced nonalcoholic fatty liver disease (NAFLD) in mice by decreasing hepatic triglyceride accumulation and suppressing pro-inflammatory cytokine expression [21]. It also alleviates alcoholic liver disease through modulating gut microbiota and host metabolism, reducing hepatic steatosis and inflammation [22]. Mechanistically, Amuc_1100 has been shown to interact with toll-like receptor 2 (TLR2), leading to enhanced intestinal barrier function and reduced endotoxemia, which in turn ameliorates liver injury [20, 23]. Furthermore, it modulates gut microbial composition [21–23].

In fish, emerging evidence suggests that Amuc_1100 exerts similar hepatoprotective effects. Surface display of Amuc_1100 on probiotic *Bacillus* strains has been shown to improve hepatic steatosis and intestinal health in HFD-fed zebrafish [24]. Recombinant Amuc_1100 expressed in *Lactococcus lactis* regulates metabolism and intestinal health in largemouth bass fed high-starch diets, reducing liver lipid deposition and alleviating hepatic inflammation [25]. These findings indicate that Amuc_1100 holds promise as a feed additive for improving liver health in farmed fish. However, the underlying mechanism by which Amuc_1100 exerts its protective role in fish remains unexplored, and the key mediators, particularly the identity of its host receptor and the role of gut microbiota, are yet to be identified.

Zebrafish (*Danio rerio*) are widely used as a teleost model in aquaculture and hepatic lipid metabolism research due to their evolutionary conservation of lipid metabolism and structural liver similarities with mammals [26–28]. They develop progressive hepatic steatosis, supporting their utility for studying lipid dysregulation [29], and the availability of germ-free (GF) zebrafish enables direct investigation of host-microbiota interactions [30]. Zebrafish are also emerging as a key system for evaluating therapeutic strategies against HFD-induced hepatic lipid accumulation [31, 32].

To elucidate the mechanism of Amuc_1100 in fish, we used 1-month-old zebrafish and larvae (5 d post-fertilization [dpf]) for complementary purposes: adults for histological and microbiota analyses, larvae for rapid screening, whole-mount Oil Red O (ORO) staining, and gene knockdown. This study demonstrates that Amuc_1100 alleviates HFD-induced hepatic lipid accumulation via a gut microbiota-dependent mechanism mediated by intestinal 14-3-3β/α-A. Amuc_1100 remodels the gut microbiota by increasing beneficial *Bacillus* and decreasing potentially harmful *Acinetobacter*, *Plesiomonas*, and *Aeromonas*. This remodeling requires 14-3-3β/α-A, a protein we identified as a direct interacting partner of Amuc_1100 and which functions as an intracellular pattern recognition receptor (PRR) with antimicrobial activity [33, 34]. Pharmacological inhibition or gene knockdown of 14-3-3β/α-A abolished Amuc_1100's effects on both microbiota and hepatic phenotype. Thus, this study establishes 14-3-3β/α-A as a key host factor in the gut-liver axis and highlights Amuc_1100 as a promising bioactive molecule. Given the evolutionary conservation of 14-3-3 proteins and lipid metabolism, these findings may also inform human metabolic health.

## Methods

### Purification of Amuc_1100

Amuc_1100 was produced according to previously reported methods with modifications [19, 20]. Briefly, the DNA encoding the Amuc_1100 (residues I31–D317, without the transmembrane domain, UniProt: B2UR41) with histidine (His)- or GFP-tag at the C-terminus was synthesized in the Sangon Biotech (Beijing, China), and cloned into the *p*ET-28a (+) vector (Novagen) with *Nde*I and *Xho*I restriction sites. Then, the plasmid *p*ET-28a (+)-Amuc_1100-His/GFP was transformed into *Escherichia coli* (*E. coli*) BL21 (DE3) (Transgen, Bejing, China). This bacterium was grown in lysogeny broth (LB) medium containing 50 μg/mL kanamycin (kana) with shaking at 200 r/min at 37 °C, followed by induction through the addition of 0.1 mmol/L isopropyl β-D-1-thiogalactopyranoside in the LB medium during the mid-exponential phase (optical density [OD] 600: 0.6–0.8). After a 16-h induction at 16 °C, bacteria were pelleted by centrifuging for 10 min at 6,880 × *g* at 4 °C. Then the pellets were resuspended using PBS and crushed by sonication at 4 °C. Supernatant was collected after centrifugation at 6,880 × *g* for 5 min at 4 °C. Amuc_1100 was purified using Ni-NTA Beads 6FF (Smart-Lifesciences, Changzhou, China). Impurities were removed with 50 mmol/L imidazole. Amuc_1100 was collected with 200 mmol/L imidazole. The concentration and purity were determined using the bicinchoninic acid method and 12% sodium dodecyl sulphate (SDS) polyacrylamide gel electrophoresis followed by Coomassie brilliant blue staining. The Amuc_1100 was stored at −80 °C until use.

### Fish husbandry

One-month-old zebrafish of the Tübingen strain were bred and maintained at the zebrafish facility of the Institute of Feed Research, Chinese Academy of Agricultural Sciences (Beijing, China). The size of each tank was 25.5 cm ×18.5 cm ×18.0 cm. The water in the rearing system was kept running; the rearing temperature was 25–28 °C, the dissolved oxygen was > 6.0 mg/L, the pH was 7.0–7.2, ammonia-N < 0.5 mg/mL, and nitrite was < 0.01 mg/L. Zebrafish were maintained at a 14:10 light-dark (L-D) cycle.

Conserved zebrafish larvae were generated at the zebrafish facility of the Institute of Feed Research, Chinese Academy of Agricultural Sciences (Beijing, China). To obtain conserved zebrafish larvae, parent zebrafish were maintained in a separate breeding system with a 14:10 L-D cycle. For egg collection, 3–4 pairs of adult fish (3–4-month old), were placed in spawning tanks with a 1:1 female-to-male ratio. A mesh insert was used to separate males and females in each spawning tank during the evening before spawning. By removing the mesh insert and turning on the light, spawning was initiated the following morning. Eggs were collected within 1–2 h. Viability of collected eggs was assessed visually. Fertilized eggs were identified by their transparent, uniform appearance and the presence of a clearly visible chorion with a perivitelline space. Unfertilized or dead eggs became opaque or abnormal shape and were removed using a sterile Pasteur pipette. Zebrafish eggs were kept in embryo medium at 28 ºC. Zebrafish larvae hatched from their chorions at 3 dpf. At 3 dpf, chorions and two-thirds of the medium were gently removed using Pasteur pipettes. Zebrafish larvae were allocated to 30-mL culture bottles at a density of 20 larvae per bottle using a Pasteur pipette, and kept in an incubator with a constant temperature of 28 °C and 14:10 L-D cycle until Exp. 2.

GF zebrafish were generated at the zebrafish facility of the Institute of Feed Research, Chinese Academy of Agricultural Sciences (Beijing, China), following an established protocol [30]. In brief, zebrafish eggs from normal parent zebrafish were disinfected with antibiotics (ABS), poly(vinylpyrrolidone)-iodine complex and sodium hypochlorite in sequence, and hatched in 30-mL culture bottles holding gnotobiotic zebrafish medium (GZM). For egg collection, 3–4 pairs of adult fish (3–4-month old), were placed in spawning tanks with a 1:1 female-to-male ratio. At 3 dpf, chorions were removed and two-thirds of the GZM were gently refreshed using Pasteur pipettes. Then GF zebrafish were kept in an incubator with a constant temperature of 28 °C and 14:10 L-D cycle until Exp. 4, Exp. 7 or Exp. 9.

One-month-old fish were acclimated for one week on a standard diet (AP100, Zeigler, USA) prior to the start of the Exp. 1, Exp. 5 or Exp. 8. Larval and GF zebrafish were not fed until 5 dpf, when exogenous feeding began.

### Experimental diets and design

This study comprises 9 independent experiments using 1-month-old zebrafish, larval zebrafish, GF zebrafish, and cell lines (zebrafish liver cells [ZFL], zebrafish embryonic fibroblast cells [ZF4], and Human Embryonic Kidney 293T [HEK293T]), each of which is described in detail as follows.

#### Main study

The feed formulation for 1-month-old zebrafish is presented in Table S1. Casein, soybean oil and lard oil, and wheat flour were used as the dietary protein, lipid and carbohydrate sources, respectively. The low-fat diet (LFD) was supplemented with soybean oil and lard at 60 g/kg diet. The HFD was supplemented with soybean oil and lard at 160 g/kg diet. Amuc_1100 at additive amounts of 25, 50, 100 or 200 mg/kg was added to HFD formulation in the form of ice-cold powder to prepare Amuc_1100-supplemented diet (AM0.0025, 0.005, 0.01 or 0.02). The supplementation of Amuc_1100 was compensated by decreasing equal levels of casein. Diet preparation was conducted according to a previous study [35]. Each diet was prepared in 50-g batches. All dry ingredients were ground through a 60-mesh screen, mixed with oil and water, extruded through a 2.5-mm aperture, dried, and stored at −20 °C. Before feeding, the pellets were ground through a 30-mesh screen.

In the Exp. 1, a zebrafish model of hepatic lipid accumulation was established through a 4-week HFD feeding. To determine the optimal dose for subsequent mechanistic studies, we evaluated the dose-dependent effects of Amuc_1100 based on body weight gain, feed conversion ratio, and hepatic triacylglycerol (TAG) content in HFD-fed zebrafish. Healthy and uniformly sized 1-month-old zebrafish (1.026 ± 0.002 g/20 fish) were randomly allocated into 6 groups and fed either an LFD, HFD, or HFD supplemented with Amuc_1100 at 25, 50, 100, or 200 mg/kg diet (AM0.0025, AM0.005, AM0.01, and AM0.02, respectively) for 4 weeks, twice a day at 9:00 and 16:00. Each group was assigned to 4 tanks (*n* = 4 per group), with 20 fish per tank. During the feeding trial, zebrafish were fed at a daily feeding rate of 6% of body weight, as recommended by a standardizing feeding management protocol in laboratory conditions [36]. At the end of the 4-week feeding trial, survival rate was recorded for each tank (100 × final survival individuals/initial individuals). Body weight gain and feed conversion ratio were calculated based on the mean values of each tank, respectively (100% × [final body weight − initial body weight]/initial body weight; feed intake/[final body weight − initial body weight]) (*n* = 4 per group).

At 12 h after the last feeding, 33 fish from each group were randomly caught, and their livers were collected for the analysis of histology (hematoxylin and eosin [H&E], ORO staining) (*n* = 4), gene expression (*n* = 3), hepatic TAG (*n* = 4) and lipopolysaccharide (LPS)-binding protein (LBP) (*n* = 4) levels. Another 16 fish from each group were sacrificed for serum alanine transaminase (Alt) and endotoxin (*n* = 4) detection. For gut microbiota analysis, 9 fish per group (2–3 fish from each of the 4 replicate tanks) were randomly sampled at 4–6 h after the last feeding. Each fish was processed individually as a biological replicate. The remaining fish were used to collect gut content for gut microbiota analysis (*n* = 9) and GF zebrafish transferring. All fish were anesthetized with tricaine methanesulfonate (MS222, 20 mg/L) before sampling.

In the Exp. 2, micro-particulated diets for zebrafish larvae were formulated as shown in Table S2, and prepared as described in a previous study [35]. All dry ingredients were ground through a 100-mesh screen. The diets were prepared by mixing the dry ingredients with the oil and water manually. Then each diet was extruded in a manual extruder with a 2.5-mm aperture. The extruded pellets were dried and stored at −20 °C in plastic bags in small quantities. Before feeding, the feed pellets were ground through a 60-mesh screen. At 5 dpf, larvae have completely absorbed their yolk sacs and rely entirely on exogenous feeding [37]. Thus, zebrafish larvae were divided into 3 groups at random at 5 dpf and fed either a LFD, HFD or AM0.01 for 7 d, twice a day at 9:00 and 16:00. Previous studies have demonstrated that a 7-d HFD intervention is sufficient to induce detectable hepatic lipid accumulation in zebrafish larvae, as confirmed by ORO staining [35, 38]. This short-term larval model allows for rapid assessment of treatment effects while minimizing potential confounding factors associated with long-term rearing, complementing the 4-week 1-month-old model used in Exp. 1. Each group was assigned to 3 bottles at a density of 20 fish per bottle (30 mL medium). Each treatment group was allocated 60 zebrafish in total. After the 7-d feeding, zebrafish larvae were collected for ORO whole-mount analysis.

#### Validation study

In the Exp. 3, we used ZFL cells to assess whether Amuc_1100 acts directly on hepatocytes. ZFL cells were seeded at a density of 1 × 10^5^ cells/well on a 6-well plate. To evaluate the direct effect of Amuc_1100 on the lipid accumulation, cells were treated for 24 h with control (no oleic acid [OA], no Amuc_1100), OA control (100 μmol/L OA, no Amuc_1100), and OA-supplemented with Amuc_1100 treatment (100 μmol/L OA with Amuc_1100 at 1, 10, or 20 μg/mL). Each group was performed in 6 independent wells (*n* = 6 per group). After 24 h post treatment, cells were harvested to evaluate intracellular TAG levels.

In Exp. 4, GF zebrafish at 5 dpf were fed the sterile micro-particulated HFD for 7 d. The 7-d feeding period was chosen for the following reasons. Firstly, GF zebrafish larvae are maintained under gnotobiotic conditions, which are technically demanding and have a higher risk of contamination during prolonged culture. Secondly, a 7-d period is sufficient for microbiota colonization and establishment, as reported in established gnotobiotic zebrafish protocols [30]. Thirdly, our previous experiments showed that 7 days of HFD feeding in GF larvae recapitulates the hepatic lipid accumulation phenotype observed in conventional larvae, validating this time point for subsequent microbiota transplant experiments [35, 38]. During the feeding period, GF zebrafish were divided into 3 groups and transferred either the gut microbiota from 1-month-old LFD-, HFD- or AM0.01-fed zebrafish at 5 dpf. Each group was assigned to 3 bottles at a density of 20 fish per bottle. Each treatment group was allocated 60 zebrafish in total. After the 7-d feeding, GF zebrafish were collected for ORO whole-mount analysis.

In Exp. 5, 1-month-old zebrafish were fed ABS-supplemented LFD, HFD, or AM0.01 for 4 weeks to deplete the gut microbiota. Polymyxin B and neomycin were added to the diets listed in Table S1. The inclusion levels of the two ABS in diets were at 0.25% and 0.33%, respectively. Healthy, uniformly sized 1-month-old zebrafish (0.9 ± 0.005 g/20 fish) were divided into 3 groups at random and fed either the ABS-supplemented LFD, HFD, or AM0.01 for 4 weeks, twice a day at 9:00 and 16:00. Each group was assigned to 3 tanks at a density of 20 fish per tank. Each treatment group was allocated 60 zebrafish in total. The maintenance was conducted as mentioned above. The primary endpoints of Exp. 5 were histological assessment and quantitative measurement of hepatic TAG, which were considered sufficient to determine whether microbiota depletion abolished Amuc_1100-mediated protection. Therefore, at 12 h after the last feeding, 6 fish from each group were sacrificed to collect livers for analysis of histology (H&E and ORO staining). Another 9 fish were used for detecting hepatic TAG levels.

In Exp. 6, fish gut-derived *Bacillus*, *Acinetobacter*, *Plesiomonas* and *Aeromonas* strains were cultured ex vivo and exposed to Amuc_1100 or a PBS control to assess the direct effect of Amuc_1100. Fish gut-derived *Bacillus*, *Acinetobacter*, *Plesiomonas* and *Aeromonas* strains were inoculated to agar or liquid medium with addition of 0.1 mg/mL Amuc_1100, and cultured ex vivo. After 18 h post incubation at 37 °C, inhibition zones on the agar surface were read for the antimicrobial testing of Amuc_1100. Meanwhile, dilution plate counting method was used to determine the effects of 0.1 mg/mL Amuc_1100 on the growth of these strains in the liquid medium. To determine the direct impact of Amuc_1100, fish gut-derived *Bacillus*, *Acinetobacter*, *Plesiomonas* and *Aeromonas* strains were cultured ex vivo and exposed to Amuc_1100 or a PBS control. These strains were transferred to GF zebrafish that fed on a sterile micro-particulated HFD or AM0.01. After the 7-d feeding, GF zebrafish were collected for bacterial DNA extraction and absolute quantification PCR (qPCR) by using primers set for specific bacterial groups targeting the 16S rDNA gene (Table S3). Next, we collected and filtered gut content supernatant from both the HFD and AM0.01 groups and added it to the culture medium. This medium was then used for the ex vivo culture of fish gut-derived strains of *Bacillus*, *Acinetobacter*, *Plesiomonas* and *Aeromonas*. Gut contents were collected and resuspended in PBS at a concentration of 25 mg/mL, followed by sterile filtration. The resulted supernatants were added to the liquid medium. After 18 h post incubation at 37 °C, dilution plate counting method was used to determine the effects of supernatants on the growth of these strains in the liquid medium.

In Exp. 7, pull-down assays using recombinant Amuc_1100 as bait were performed to identify its interacting host proteins. Ten healthy 1-month-old zebrafish fed the LFD were sacrificed to collect intestines for identifying interacting proteins using pull-down and liquid chromatography-mass spectrometry (LC-MS) methods. Recombinant His-tagged Amuc_1100 was immobilized to the Pierce Spin Colum and incubated with intestinal lysates. Bound proteins were eluted with imidazole and identified by LC-MS/MS. This unbiased approach was chosen to capture constitutive binding partners under basal physiological conditions. Candidate interacting proteins were validated by co-immunoprecipitation (co-IP) in HEK293T cells. To assess cellular uptake, zebrafish ZF4 cells were incubated with Amuc_1100-GFP and observed by fluorescence microscopy. To predict potential interaction domains, Amuc_1100 was subjected to simulated intestinal digestion, and stable fragments were analyzed by LC-MS/MS. The 3D structure of Amuc_1100 was predicted using DMFold, and interaction sites were visualized with Mol* 3D Viewer (Protein Data Bank). Potential candidate binding motifs were identified based on solvent accessibility and surface complementarity.

In Exp. 8, we treated 1-month-old zebrafish with BV02 to determine whether 14-3-3β/α-A mediates the effect of Amuc_1100 on gut microbiota. BV02 is a well-characterized inhibitor of 14-3-3 protein-protein interaction (PPI) that has been validated by nuclear magnetic resonance (NMR) spectroscopy [39] and functional studies [40, 41]. It was added to the diets that listed in Table S1. BV02 acts by competitively binding to the client-binding pocket, thereby disrupting the interaction between 14-3-3 and its client proteins [41]. The inclusion level of BV02 in diets was at 0.017%. This experiment followed the same design as Exp. 1. Healthy and uniformly sized 1-month-old zebrafish (1.56 ± 0.001 g/20 fish) were divided into 6 groups at random and fed either the LFD-BV02, HFD-BV02, or AM0.01-BV02 diet for 4 weeks, twice a day at 9:00 and 16:00. Each group was assigned to 3 tanks at a density of 20 fish per tank. Each treatment group was allocated 60 zebrafish in total. The maintenance was conducted as mentioned above. At 12 h after the last feeding, 6 fish from each group were sacrificed for analysis of histology (H&E and ORO staining). Another 9 fish were used for detecting hepatic TAG levels. Additional 9 fish were used for detecting hepatic LBP levels. At 4–6 h after the last feeding, the remaining fish were used to collect gut content for gut microbiota analysis and GF zebrafish transferring.

In Exp. 9, zebrafish larvae at 5 dpf were divided into 3 groups, and fed either a micro-particulated LFD, HFD, or AM0.01 for 7 d, twice a day at 9: 00 and 16: 00. During feeding period, *14-3-3β/α-A* vivo-morpholino oligonucleotides (vivo-MO) was added to the medium to reduce the expression of *14-3-3β/α-A*. After the 7-d feeding, zebrafish larvae were collected for ORO whole-mount analysis.

### Histology

Livers were rinsed with PBS buffer and fixed in 4% paraformaldehyde. For H&E staining, livers were collected from 4 fish per group. Each liver was embedded and sectioned at 5–8 μm thickness for H&E staining and 8–10 μm for ORO staining. After dewaxing using xylene twice for 20 min and rehydration, hematoxylin and eosin were used to stain nucleus and cytoplasm respectively. Following dehydration by 75%, 85%, 95% and 100% ethanol in sequence and being transparent by xylene twice for 2 min. For ORO staining, livers were embedded in optimal cutting temperature compound and cut into 8–10 μm sections. Liver sections were washed three times for 15 min, followed by rinses in 60% isopropanol for 5 min. Then liver sections were stained by 0.5% ORO solution in 100% isopropanol for 15 min and washed with distilled water, followed by counterstaining with hematoxylin. Images of H&E and ORO staining were captured on a Digital slice scanning system (KF-PRO-120, Ningbo, China). At least 2 non-consecutive sections per liver were prepared, and three random high-power fields (400 × magnification) per section were examined under a light microscope. All sections were evaluated independently and blindly.

For ORO whole-mount analysis, sample sizes were determined based on power analysis using pilot data. Whole larvae were fixed with 4% paraformaldehyde, washed with PBS buffer, infiltrated with a graded series of propylene glycol baths, and stained with 0.5% ORO in 100% propylene glycol overnight. The stained larvae were washed with decreasing concentrations of propylene glycol, followed by several rinses with PBS, and stored in 20% propylene glycol bath. Images of ORO whole-mount analysis of larvae were obtained using a microscope (Zeiss SteREO Discovery.V8, Germany). For the quantification of ORO whole-mount analysis of zebrafish larvae, images were converted to the 8-bit gray scale for measuring mean gray value using ImageJ software. The intensity of images was quantified using ImageJ and used to quantitatively evaluate steatosis.

### Detection of serum Alt

Serum of zebrafish was collected as previously described [42]. Briefly, blood samples were collected in tubes from the zebrafish following cutting off the tail. Serum was then separated by centrifugation at 1,467 × *g* for 10 min. The activity of Alt was detected using a commercial kit (Nanjing Jiancheng Bioengineering Institute, Jiangsu Province, China). Serum and gradient diluted standards (sodium pyruvate) reacted with the substrate composed of alanine and α-ketoglutaric acid at 37 °C for 30 min, followed by incubation with 2,4-dinitrophenylhydrazine for 20 min. Phenylhydrazine products were examined under in alkaline condition at 510 nm. Alt activity was calculated according to the standard curve and expressed as enzyme activity units per liter (U/L).

### Cell culture

The ZFL (ATCC^®^ CRL-2643™), ZF4 (ATCC^®^ CRL-2050™) and HEK293T (ATCC^®^ CRL-3216™) cell lines were purchased from American Type Culture Collection (Manassas, VA, USA), and cultured according to an established protocol [43, 44]. All media were obtained from Corning Inc. Penicillin-Streptomycin solution and bovine insulin were purchased from Sigma (St. Louis, MO, USA). Murine epidermal growth factor was purchased from Peprotech (Rocky Hill, NJ, USA). Rainbow trout (*Oncorhynchus mykiss*) serum was purchased from Caisson Labs (USA). ZFL and ZF4 cells were maintained at 28 °C in a humidified 5% CO_2_ and 95% air atmosphere. HEK293T cells were maintained at 37 °C in a humidified 5% CO_2_ and 95% air atmosphere.

### Detection of TAG

To detect the intracellular TAG, cells were lysed using 1% SDS buffer, followed by ultrasonic crush at 4 ℃. The n-hexane:isopropanol (3:2) solution was added to cell lysates. After vigorous vortex for 1 min, cell lysates were centrifuged at 175 × *g* for 15 min to separate the phases. The lipid-containing up-phase was carefully aspirated. To detect the hepatic TAG, livers were homogenized in PBS at 4 °C. Then homogenates were vortexed with the chloroform:methanol (2:1) solution for 1 min. After standing for 10 min, the lipid-containing bottom-phase was extracted using a metal needle. The lipid-containing phases were then evaporated in a 70 °C metal bath with nitrogen stream. The dried lipids were emulsified in chloroform containing 1% Triton ×100. Then emulsified lipids were dried at 70 °C with nitrogen stream again and reconstituted in distilled water. The TAG levels were determined by a quantitative enzymatic method as described in a previous study [45]. The protein concentration of cells was determined by using a Bradford reagent (Sigma, St. Louis, MO, USA). Intracellular TAG levels were normalized to the protein amounts of each sample (µg/mg protein). Hepatic TAG levels were normalized to the liver weight (µg/mg tissue).

### Detection of serum endotoxin by LAL test

Serum endotoxin levels were determined using the ToxinSensor™ Chromogenic LAL Endotoxin Assay Kit (Genscript, Jiangsu Province, China) according to the manufacturer’s instructions. In brief, serum samples were dispensed into endotoxin-free vials, and then incubated with LAL and chromogenic substrate consecutively. After incubation, stop buffer was added to each reaction vial. The absorbance of each sample was measured using the SynergyMX Multi-Functional MPP Detector (Biotek, USA) at 545 nm. The serum level of endotoxin in adult zebrafish was expressed as endotoxin units per milliliter (EU/mL).

### Detection of LBP

The levels of LBP in the livers were determined using an ELISA kit (MLBio, Shanghai, China). Livers were weighted and homogenated in pre-cooled PBS, followed by centrifuging at 13,201 × *g* for 5 min at 4 °C to get the supernatant. Adding 100 μL diluted supernatants into the antibody-coated plate. After incubation for 90 min at 37 °C, discarding the liquid from each well, and adding 100 μL of Biotinylated Detection Ab working solution to each well immediately. After an incubation at 37 °C for 1 h, discarding the solution and adding 350 μL of wash buffer to rinse each well for 1 min. Then, horseradish peroxidase (HRP)-conjugated working solution was added to each well and incubated for 30 min at 37 °C. Discarding the solution, and adding 90 μL of substrate reagent and 50 μL of stop solution to each well in sequence. The OD of each well was determined at the wavelength of 450 nm. The level of LBP was expressed as the molar weight of LBP per gram protein (μmol/g protein).

### Gene knockdown

For zebrafish larvae, gene knockdown was conducted by using vivo-MO synthesized by Gene Tools (Philomath, OR, USA). The sequences of MO used in this study are as follows: *14-3-3β/α-A *vivo-MO, 5′-AACATCGAGTATCTCACCAGCACG-3′; and standard control-MO (std-MO), 5′-CCTCTTACCTCAGTTACAATTTATA-3′. At 5 dpf, zebrafish larvae were immersed with 50 nmol/L std-MO or *14-3-3β/α-A *vivo*-*MO for 7 d. The efficiency of both the vivo-MO was determined by qPCR.

### Bacterial 16S ribosomal RNA (rRNA) sequencing

The E.Z.N.A. stool DNA kit (Omega Biotek, Norcross, GA, USA) was used to extract bacterial DNA. The 16S ribosomal DNA (rDNA) V3–V4 regions were amplified using 341 F (CCTACGGGNGGCWGCAG) and 806R (GGACTACHVGGGTATCTAAT) as primers. The PCR products were purified and then sequenced by an Illumina MiSeq platform (Guangzhou Gene Denovo Co., Ltd., Guangzhou, China). Raw sequencing data were processed using QIIME2 (version 2024.10). The raw sequences were screened by removing the sequences containing over 10% unknown nucleotides (N) and < 80% of bases with Q value > 2 [46]. Paired-end clean reads were merged as raw tags using FLSAH (v 1.2.11) with a minimum overlap of 10 bp and mismatch error rates of 2%. Clean tags were searched against the reference database (http://drive5.com/uchime/uchime_download.html) to perform Reference-based chimera checking using UCHIME algorithm (http://www.drive5.com/usearch/manual/uchime_algo.html). The effective tags were clustered into operational taxonomic units (OTU) at 97% similarity using the UPARSE pipeline. Following OTU determination, the gut microbiota indices were assessed using the Omicsmart platform (Gene Denovo Biotechnology Co., Ltd., Guangzhou, China; https://www.omicsmart.com).

### Ex vivo culturing of bacteria

Fish gut-derived *Bacillus*, *Acinetobacter*, *Plesiomonas*, *Aeromonas* strains were cultured in Amuc_1100 or gut content supernatants-supplemented medium, and incubated at the optimal temperature for 18 h, respectively. Dilution plate counting method was used to determine the effects of Amuc_1100 or gut content supernatants on the growth of these strains.

### Antimicrobial testing

Fish gut-derived *Acinetobacter*, *Plesiomonas* and *Aeromonas* strains were inoculated evenly over the agar surface. Then disks loaded with PBS or Amuc_1100 (0.1 mg/mL) were applied firmly on the agar surface. After 18 h post incubation at 37 °C, inhibition zones were read for antimicrobial assessment of Amuc_1100.

### GF zebrafish feeding and gut microbiota transfer

Zebrafish larvae complete absorption of yolk by 5 dpf and can be subjected to feeding and gut microbiota transfer trials [37]. Sterilized micro-particulated diets were generated by irradiation with 20 kGy gamma ray (Beijing Hongyisifang Radiation Technology Co., Ltd., Beijing, China). At 5 dpf, GF zebrafish began consuming the sterilized HFD until 12 dpf. During feeding period, gut microbiota was transferred into these GF zebrafish according to a reported protocol [30]. The bacterial concentration for transplantation was determined based on published literature [47] and empirical data. The microbiota load in the intestine of conventional zebrafish is approximately 10^8^ colony forming units (CFU)/mg gut content [47]. In our preliminary experiments, each 1-month-old zebrafish yielded 2–5 mg of gut content (wet weight), corresponding to an estimated bacterial load of 2–5 × 10^8^ CFU/fish. Gut contents from ten donor fish were pooled and resuspended in 4 mL of sterile GZM, yielding an estimated concentration of approximately 5–12.5 × 10^8^ CFU/mL. The bacteria were added to the sterilized medium containing 5 dpf GF zebrafish at a 1:100 (v/v) ratio, resulting in a final concentration of 5–12.5 × 10^6^ CFU/mL in the larval medium. To validate this estimate, 100 μL of medium was collected immediately after inoculation, serially diluted, and spread onto LB agar plates. Colonies were counted after 24 h incubation at 28 °C, confirming that the actual concentration fell within the range of 1–5 × 10^6^ CFU/mL. At 12 dpf, zebrafish larvae were collected for ORO staining.

### Bacterial DNA extraction and absolute quantification

The number of a specific bacterial groups was quantified using absolute qPCR as described in a previous study [38, 48]. Primer sets for specific bacterial groups targeted the 16S rDNA gene are listed in Table S3. For the zebrafish larvae, results were expressed as Log10 copy numbers of bacterial 16S rDNA per fish.

### Affinity pull-down and LC-MS analysis

Pull-down was performed following the instructions of a His-tagged protein interaction pull-down kit (Pierce, Rockford, USA). Firstly, recombinant His-tagged Amuc_1100 served as the bait protein and was immobilized to the Pierce Spin Column. Whole protein from zebrafish intestines served as the prey proteins. Adding prepared prey proteins into the spin column containing the immobilized His-tagged Amuc_1100, which was then incubated at 4 °C for 12 h with gentle rocking motion on a rotating platform. Bait-prey complexes were obtained after the elution by a gradient of 200 to 500 mmol/L imidazole. The resulting bait-prey complexes were digested using MS-grade trypsin (Pierce) at 37 °C overnight. Tryptic peptides were further purified by ZipTip (Millipore, ZTC18S096) and subjected to LC-MS/MS analysis on a Q Exactive mass spectrometer (ThermoFisher Scientific). Tandem MS data was extracted by the Proteome Discoverer software (Thermo Fisher Scientific, version 1.4.0.288) with the MASCOT searching engine version 2.3.02. The database used is the zebrafish UniProtKB/Swiss-Prot database (Release 2017-11-30, with 44,128 sequences).

### Immunoprecipitation and Western blotting

The genes encoding Amuc_1100 and full-length zebrafish 14-3-3β/α-A were cloned into flag-, or HA-tagged *p*cDNA3.1 vector. HEK293T cells were transfected with *p*cDNA3.1-Amuc_1100-flag and *p*cDNA3.1-14-3-3β/α-A-HA plasmids using Lipofectamine™ 3000 Transfection Reagent (Thermo Fisher Scientific, Life Technologies). At 24 h post transfection, HEK293T cells were lysed in ice-cold buffer composed of 50 mmol/L Tris-HCl (pH 7.4), 150 mmol/L NaCl, 0.1% Nonidet (NP-40), and protease inhibitors. Lysates were incubated with anti-flag M2 beads for 12 h at 4 ℃ and then washed three times with ice-cold buffer. Protein complexes were separated by SDS-PAGE. A polyvinylidene difluoride (PVDF) membrane was used for Western blotting. After blocking nonspecific binding with 5% skimmed milk in tris buffered saline with Tween 20, the PVDF membrane was incubated with primary antibodies against flag M2-tag (CST, 14793S, 1:1,000) and HA-tag (CST, 3724S, 1:1,000). The blots were developed using HRP-conjugated secondary goat anti-rabbit (CWBIO, 0103S, 1:1,000) and detected by the electrochemiluminescence-plus system.

### Total RNA extraction, reverse transcription, and qPCR

Total RNA extraction and reverse transcription were conducted as described previously [49]. In brief, total RNA was isolated using Trizol reagent (Cwbio, Beijing, China) and then reversed transcribed to cDNA by FastKing gDNA Dispelling RT SuperMix (Tiangen, Beijing, China). The qPCR was performed using SYBR^® ^Green Supermix according to the manufacturer’s instructions (Tiangen, Beijing, China). Thermal cycling conditions were as follows: 95 °C for 10 min, followed by 45 cycles of 95 °C for 15 s, 60 °C for 30 s and 72 °C for 30 s. Melting curve analysis was performed at the end of each run (95 °C for 15 s, 60 °C for 60 s, then ramped to 95 °C at 0.11 °C/s) to verify primer specificity. Lipid anabolism-related genes encoding peroxisome proliferator-activated receptor gamma (*pparγ*), sterol regulatory element binding transcription factor 1 (*srebf1*), CCAAT/enhancer-binding protein alpha (*c/ebpα*), fatty acid synthase (*fas*), acetyl-CoA carboxylase 1 (*acc1*), and diacylglycerol acyltransferase 2 (*dgat2*), lipid catabolism-related genes encoding adipose triglyceride lipase (*atgl*) and carnitine palmitoyltransferase 1Aa (*cpt1aa*), and *14-3-3β/α-A* were analyzed. Reference gene encoding ribosomal protein S11 (*rps11)* was used as the internal control. All primer pairs used in this study produced a single, sharp peak in the melting curve, confirming the absence of non-specific amplification or primer-dimer formation. The qPCR primers used, including their sequences and amplicon sizes, are listed in Table S3. The results were stored, managed, and analyzed using LightCycler 480 software (Roche, Basel, Switzerland). Relative gene expression was calculated using the 2^-ΔΔCt^ method, with *rps11* as the reference gene.

### Data analysis

The statistical analyses were conducted using GraphPad Prism 8 software (GraphPad Software Inc., San Diego, CA, USA). Results are expressed as the mean ± standard errors of the mean (SEM). Comparisons between two groups were analyzed using the Student’s *t*-test, and comparisons between multiple groups were analyzed using one-way ANOVA followed by a Duncan’s test. The statistical significance was set at *P* < 0.05.

## Results

### Amuc_1100 alleviates hepatic lipid accumulation induced by HFD in zebrafish

**Fig. 1:**
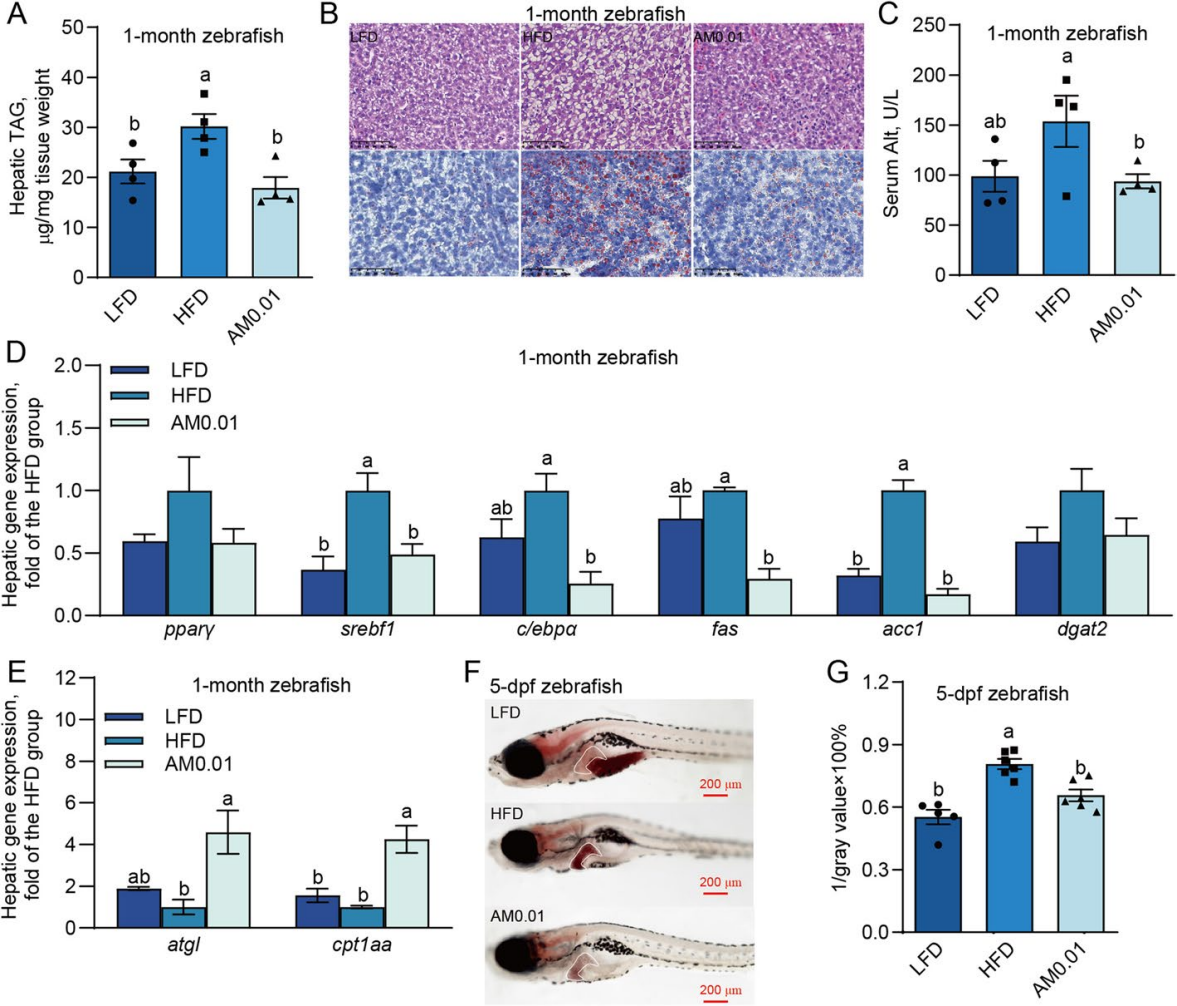
Amuc_1100 alleviates the hepatic lipid accumulation induced by the HFD. **A**−**G** One-month-old zebrafish fed on the LFD, HFD and AM0.01 for 4 weeks. **A** Hepatic TAG contents (*n* = 4).** B** Representative images of H&E- and ORO-stained liver sections. The scale bar = 50 μm. **C** Serum ALT activity (*n* = 4). **D** and **E** The relative mRNA expression of (**D**) lipid anabolism-related genes (*pparγ*, *srebf1*, *c/ebpα*, *fas*, *acc1* and *dgat2*) and (**E**) lipid catabolism-related genes (*atgl* and *cpt1aa*) in the liver (*n* = 3).** F** and **G** Zebrafish at 5 dpf fed on the LFD, HFD and AM0.01 for 7 d. **F** Representative images of whole-mount ORO staining. The scale bar = 200 μm. **G** Quantitative assessment of whole-mount ORO staining (*n* = 5 or 6). Data are expressed as mean ± SEM. Means without a common letter are significantly different (*P* < 0.05). Duncan’s test

The HFD-fed zebrafish showed a survival rate comparable to those fed the LFD (100% in all groups), but with significantly increased body weight gain (83.59% vs. 67.29%; *P* < 0.05) and a tendency of reduced feed conversion ratio (1.24 vs. 1.55; *P* = 0.052) (Fig. S1A–C) at the end of a 4-week feeding trial. Hepatic TAG content was significantly elevated in the HFD group (30.17 μg/mg tissue) compared to the LFD group (21.19 μg/mg tissue; *P* < 0.05) (Fig. S1D; Fig. 1A). Among the 4 doses tested (25, 50, 100, and 200 mg/kg diet), all Amuc_1100-supplemented groups showed a trend toward reduced body weight gain, increased FCR and lowered hepatic TAG levels (Fig. S1A–D). The 100 mg/kg dose exhibited the most pronounced reduction in hepatic TAG accumulation, bringing it to a level statistically indistinguishable from the LFD group (17.91 μg/mg tissue; *P* < 0.05) (Fig. S1D; Fig. 1A), while also significantly attenuating HFD-induced weight gain (Fig. S1B). The higher dose (200 mg/kg) did not confer additional benefits, suggesting a plateau in efficacy. Consistently, H&E and ORO staining revealed prominent vacuole-like degeneration in the HFD group (Fig. 1B). Therefore, the dose of 100 mg/kg was selected for all subsequent experiments investigating the mechanisms underlying Amuc_1100-mediated alleviation of hepatic lipid accumulation.

Furthermore, serum Alt activity was significantly lower in the AM0.01 group than in the HFD group (*P* < 0.05; Fig. 1C). The mRNA levels of lipid anabolism-related genes, *srebf1* and *acc1*, were significantly upregulated in the HFD group compared with the LFD group (*P* < 0.05; Fig. 1D). In contrast, these 2 genes, along with *c/ebpα* and *fas*, were downregulated in the AM0.01 group relative to the HFD group (*P* < 0.05; Fig. 1D). Meanwhile, the expression of *pparγ* and *dgat2* showed a non-significant decreasing trend (*P* > 0.05; Fig. 1D). Additionally, the mRNA levels of lipid catabolism-related genes, *atgl* and *cpt1aa*, were significantly higher in the AM0.01 group than in the HFD group (*P* < 0.05; Fig. 1E).

After 7 days of HFD feeding, zebrafish larvae showed evident red staining in the liver by ORO staining (Fig. 1F). Quantification using ImageJ revealed a significantly higher mean gray value in the liver area of the HFD group compared to the LFD group (*P* < 0.05; Fig. 1G), indicating that short-term HFD feeding (7 d) elevated intrahepatic lipid levels in zebrafish larvae. However, administration of Amuc_1100 at an additive amount of 0.01% significantly reduced HFD-induced intrahepatic staining and the mean gray value in zebrafish larvae (*P* < 0.05; Fig. 1F and G). Collectively, these findings indicate that Amuc_1100 protects zebrafish from diet-induced hepatic lipid accumulation and associated liver damage. Consistent with in vivo findings, OA exposure significantly elevated TAG content in ZFL cells (*P* < 0.05; Fig. S1E), addressing whether Amuc_1100 acts directly on hepatocytes. However, supplementation with Amuc_1100 did not reduce the TAG content in vitro (Fig. S1E), suggesting that its lipid-lowering effect may not act directly on the liver.

###  Amuc_1100 alleviates hepatic lipid accumulation via the gut microbiota in zebrafish

**Fig. 2:**
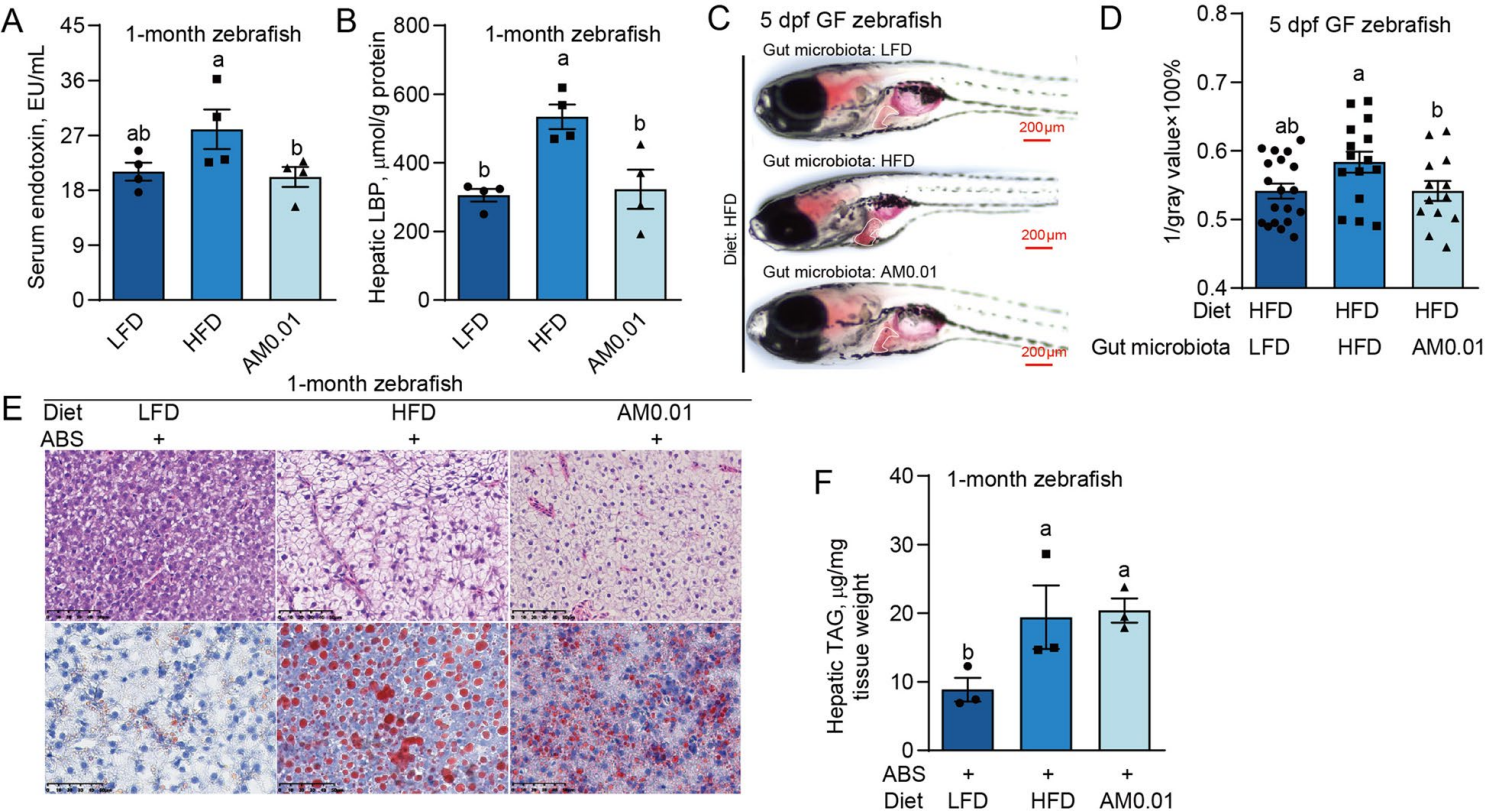
Amuc_1100-induced gut microbiota reduces HFD-induced hepatic lipid accumulation. **A** and **B** One-month-old zebrafish fed on the LFD, HFD and AM0.01 for 4 weeks. **A** Serum endotoxin levels (*n* = 4). **B** Hepatic LBP levels. (*n* = 4). **C** and **D** GF zebrafish at 5 dpf fed the HFD for 7 d, and were inoculated with gut microbiota from one-month-old zebrafish fed the LFD, HFD or AM0.01. **C** Representative images of whole-mount ORO staining. The scale bar = 200 μm. **D** Quantitative assessment of whole-mount ORO staining (*n* ≥ 13). **E** and **F** One-month-old zebrafish fed on the ABS-supplemented LFD, HFD or AM0.01 for 4 weeks. **E** Representative images of H&E- and ORO-stained liver sections. The scale bar = 50 μm. **F** Hepatic TAG contents (*n* = 3). Data are expressed as mean ± SEM. Means without a common letter are significantly different (*P* < 0.05). Duncan’s test

Compared to the HFD group, the levels of serum endotoxin and hepatic LBP were significantly reduced by Amuc_1100 administration (*P* < 0.05; Fig. 2A and B), suggesting the occurrence of microbial translocation into the liver in conventional zebrafish. In GF zebrafish, the administration of the AM0.01 did not reduce intrahepatic staining or the mean gray value compared with the HFD (Fig. S2A and B). GF zebrafish transplanted with microbiota from AM0.01-fed donors displayed markedly lighter intrahepatic staining relative to those receiving HFD-derived microbiota, consistent with attenuated hepatic lipid deposition (Fig. 2C). The quantitative assessment of whole-mount ORO staining performed with ImageJ software corroborated these morphological observations, revealing a statistically significant decrease in the mean gray value for the AM0.01 microbiota group (*P* < 0.05; Fig. 2D). After gut microbiota depletion by ABS, the protective effect of Amuc_1100 against hepatic lipid deposition was abrogated, as evidenced by comparable histological appearance between the ABS-HFD and ABS-AM0.01 groups (*P* > 0.05) (Fig. 2E). Consistently, hepatic TAG levels showed no significant difference between the 2 groups (*P* > 0.05) (Fig. 2F). This demonstrates that the gut microbiota is essential for Amuc_1100's efficacy.

### Amuc_1100 alters gut microbiota composition in zebrafish

**Fig. 3:**
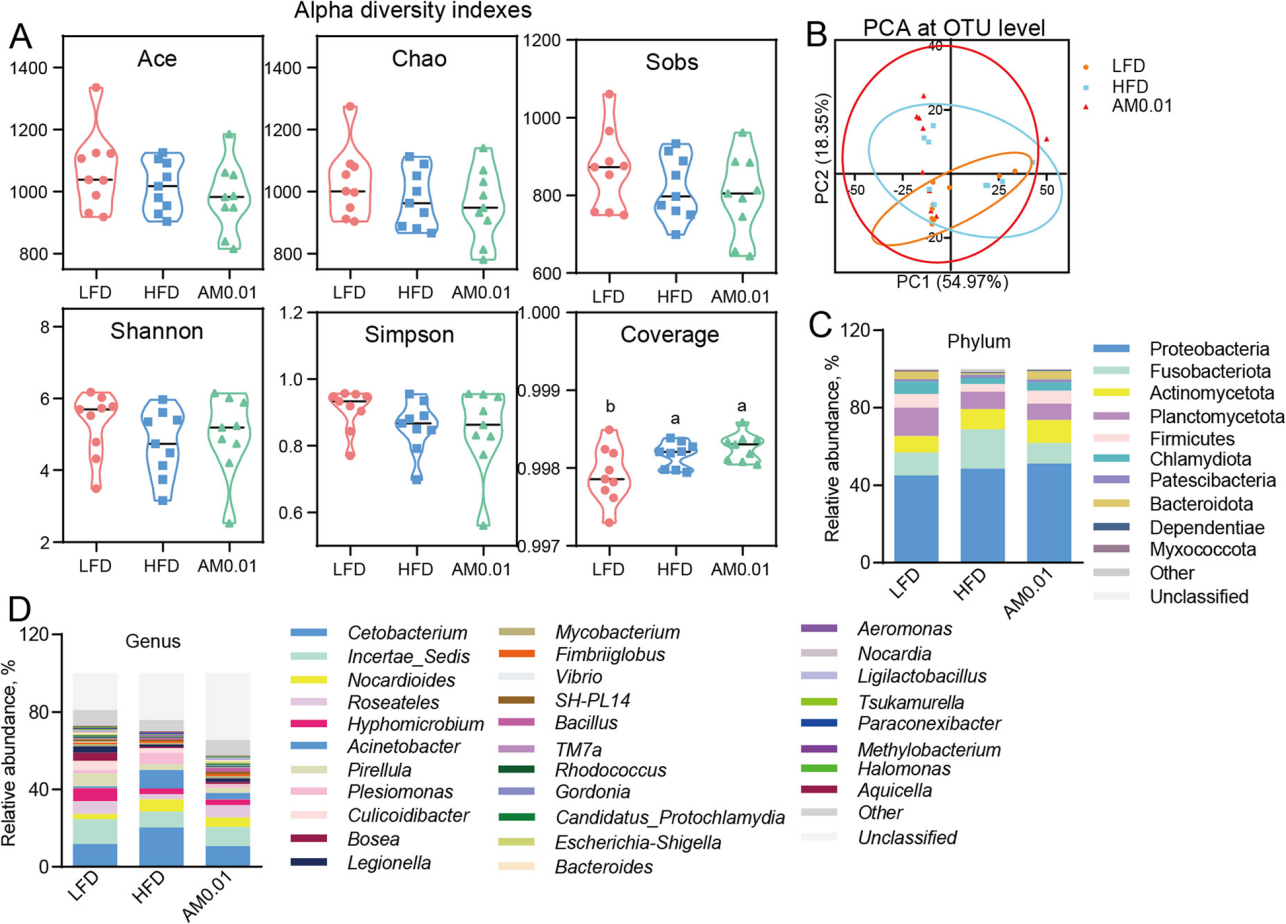
Amuc_1100 changes gut microbiota composition. **A**−**D** One-month-old zebrafish fed the LFD, HFD and AM0.01 for 4 weeks. **A** Indexes of alpha diversity (*n* = 9). **B** PCA analysis of gut microbiota at the OTU level (*n* = 9). **C** Relative abundance of gut microbial composition at the phylum level (*n* = 9). **D** Relative abundance of gut microbial composition at the genus level (*n* = 9). Data are expressed as mean ± SEM. Means without a common letter are significantly different (*P* < 0.05). Duncan’s test

In 1-month-old zebrafish, the HFD significantly altered gut microbiome composition compared to the LFD (Fig. 3A–D). However, no significant difference in alpha diversity indices was observed between the HFD and AM0.01 groups (Fig. 3A). Principal component analysis (PCA) based on Bray-Curtis distances revealed that PC1 and PC2 explained 54.97% and 18.35% of the total variance, respectively (Fig. 3B). Permutational multivariate analysis of variance (PERMANOVA) confirmed that both HFD and AM0.01 groups had significantly different gut microbiota compositions compared to the LFD group (*P* < 0.05 for both comparisons) (Fig. S3A and B). The difference between HFD and AM0.01 groups did not reach statistical significance, although a trend toward separation was observed (*P* = 0.061) (Fig. S3C). This pattern is consistent with the PCA visualization, where HFD and AM0.01 groups partially overlap but both are displaced from LFD along PC1. The gut microbiota composition of HFD-fed zebrafish was characterized as 48.5% Proteobacteria, 20.3% Fusobacteriota and 10.4% Actinomycetota at the phylum level. The abundances of Firmicutes and Proteobacteria did not differ among the 3 groups (Fig. 3C; Table 1). However, the AM0.01 group showed significant changes in bacterial abundances compared to the HFD group at genus level. The abundance of *Bacillus* increased from 0.6% to 2.0%, whereas the abundances of *Acinetobacter*, *Plesiomonas*, and *Aeromonas* decreased from 9.58% to 3.34%, 5.66% to 1.35%, and 0.540% to 0.0853%, respectively (*P* < 0.05) (Fig. 3D; Table 2). Besides, the relative abundance of Bacteroidota was 4.5-fold higher in the AM0.01 group than in the HFD group, increasing from 0.793% to 4.41% (*P* < 0.05) (Fig. 3C; Table 1). Within the phylum Bacteroidota, the abundance of the genus *Bacteroides* was 8.0-fold greater in the AM0.01 group (0.827%) than in the HFD group (0.0933%) (*P* < 0.05) (Fig. 3D; Table 2).

**Table 1:** The relative abundance of main phyla in the intestinal microbiota of zebrafish fed the LFD, HFD or AM0.01

Phylum	LFD	HFD	AM0.01
Proteobacteria	45.0 ± 5.30	48.5 ± 5.11	51.1 ± 4.48
Fusobacteriota	11.7 ± 5.36	20.3 ± 6.77	10.6 ± 7.59
Actinomycetota	8.57 ± 1.32	10.4 ± 2.18	11.9 ± 2.60
Planctomycetota	14.8 ± 3.99	9.08 ± 2.42	8.39 ± 1.53
Firmicutes	7.14 ± 2.68	3.85 ± 1.38	6.76 ± 1.82
Chlamydiota	6.32 ± 2.22	3.27 ± 0.75	4.28 ± 1.22
Patescibacteria	1.12 ± 0.34	1.61 ± 0.49	1.51 ± 0.33
Bacteroidota	3.94 ± 1.82^a^	0.793 ± 0.23^b^	4.41 ± 1.55^a^
Dependentiae	0.226 ± 0.05	0.423 ± 0.12	0.489 ± 0.09
Myxococcota	0.634 ± 0.28^a^	0.0678 ± 0.02^b^	0.0655 ± 0.01^b^

Values are expressed as the mean ± SEM (*n* = 9). Means marked with different letters differ significantly (*P* < 0.05), whereas those sharing a common letter do not. Duncan’s test

**Table 2:** The relative abundance of main genus in the intestinal microbiota of zebrafish fed the LFD, HFD or AM0.01

Genus	LFD	HFD	AM0.01
*Cetobacterium*	11.7 ± 5.72	20.3 ± 6.77	10.6 ± 7.59
*Incertae_Sedis*	13.0 ± 2.81	8.33 ± 1.62	9.87 ± 1.22
*Roseateles*	6.63 ± 2.38	2.69 ± 0.40	6.38 ± 1.24
*Acinetobacter*	0.98 ± 0.18^b^	9.58 ± 2.86^a^	3.34 ± 1.14^b^
*Pirellula*	6.72 ± 2.29	3.14 ± 0.72	2.83 ± 0.52
*Plesiomonas*	1.73 ± 0.66	5.66 ± 2.81	1.35 ± 0.49
*Culicoidibacter*	4.79 ± 2.76	2.60 ± 1.47	0.525 ± 0.18
*Bosea*	4.10 ± 2.51	0.722 ± 0.14	1.03 ± 0.32
*Legionella*	3.40 ± 1.30	1.13 ± 0.24	1.82 ± 0.65
*Fimbriiglobus*	0.923 ± 0.28	0.955 ± 0.32	1.00 ± 0.20
*SH-PL14*	1.05 ± 0.29	0.770 ± 0.20	1.04 ± 0.20
*Bacillus*	0.318 ± 0.08^b^	0.573 ± 0.23^b^	1.99 ± 0.47^a^
*TM7a*	0.324 ± 0.11	0.399 ± 0.12	0.503 ± 0.13
*Gordonia*	0.326 ± 0.11	0.0937 ± 0.02	0.438 ± 0.17
*Candidatus_Protochlamydia*	0.395 ± 0.17	0.106 ± 0.03	0.774 ± 0.63
*Bacteroides*	1.03 ± 0.48^a^	0.0933 ± 0.04^b^	0.827 ± 0.30^a^
*Aeromonas*	0.135 ± 0.06^ab^	0.540 ± 0.24^a^	0.0853 ± 0.03^b^
*Nocardia*	0.214 ± 0.09	0.0936 ± 0.05	0.0867 ± 0.03
*Ligilactobacillus*	0.578 ± 0.24	0.102 ± 0.03	0.878 ± 0.39
*Methylobacterium*	0.495 ± 0.18	0.556 ± 0.17	0.121 ± 0.02
*Halomonas*	0.469 ± 0.16	0.200 ± 0.03	0.362 ± 0.06

Values are expressed as the mean ± SEM (*n* = 9). Means marked with different letters differ significantly (*P* < 0.05), whereas those sharing a common letter do not. Duncan’s test

### The ability of Amuc_1100 to alter the zebrafish gut microbiota depends on host factors

**Fig. 4:**
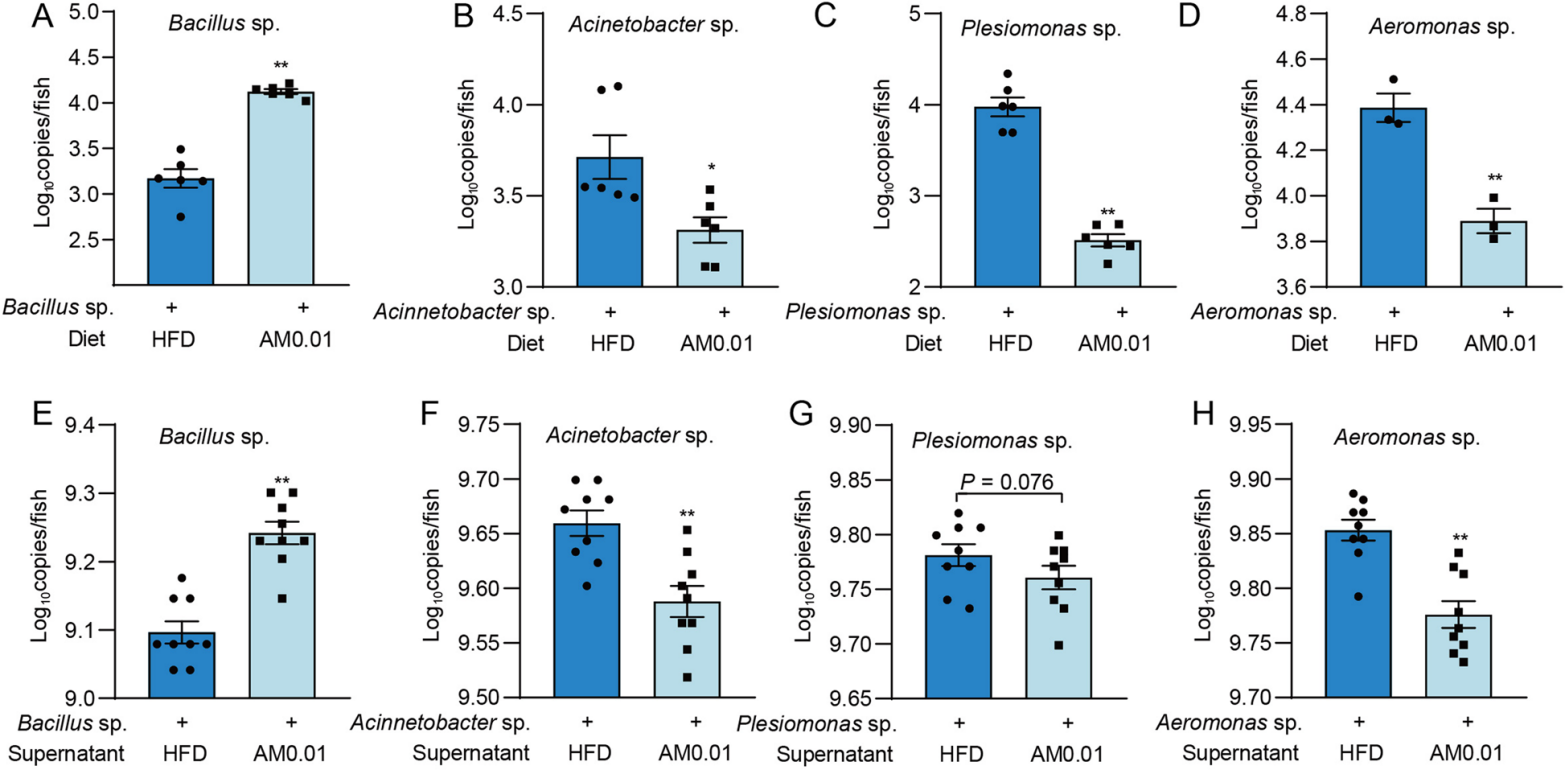
Amuc_1100 and gut content supernatant from Amuc_1100-fed zebrafish alter growth of fish gut-derived strains ex vivo. **A**−**D** The number of fish gut-derived (**A**) *Bacillus*, (**B**) *Acinetobacter*, (**C**) *Plesiomonas* and (**D**) *Aeromonas* strains after transferring to 5 dpf AM0.01-fed GF zebrafish for 7 d (*n* = 3 or 6). **E**−**H** The concentration of fish gut-derived (**E**) *Bacillus*, (**F**) *Acinetobacter*, (**G**) *Plesiomonas* and (**H**) *Aeromonas* strains after culturing in gut content supernatant-supplemented medium for 18 h (*n* = 9). Data are expressed as mean ± SEM. ^*^P < 0.05, ^**^P < 0.01. Student’s *t*-test

In GF zebrafish colonized with gut-derived *Bacillus*, Amuc_1100 feeding resulted in a markedly higher abundance of this strain than HFD feeding (*P* < 0.05; Fig. 4A). In GF zebrafish colonized with gut-derived *Acinetobacter*, *Plesiomonas*, or *Aeromonas* following a 7-d feeding period, the abundance of each bacterium was lower in the AM0.01 group relative to the HFD group (*P* < 0.05; Fig. 4B–D). In the presence of Amuc_1100, the number of the fish gut-derived *Bacillus* strain that cultured ex vivo was significantly higher (1.11 × 10^8^ CFU/mL) compared to the control (6.37 × 10^7^ CFU/mL) (*P* < 0.05; Fig. S4A). These results suggest that Amuc_1100 directly promotes the growth of *Bacillus*. However, Amuc_1100 supplementation did not alter the number of *Acinetobacter*, *Plesiomonas* and *Aeromonas* strains ex vivo, which remained at 5.50 × 10^9^, 2.31 × 10^9^, and 1.21 × 10^10^ CFU/mL, similar to the PBS control (5.07 × 10^9^, 2.14 × 10^9^, and 1.05 × 10^10^ CFU/mL) (*P* > 0.05; Fig. S4B–D). Meanwhile, no inhibition zone was observed around the PBS or Amuc_1100-loaded filter papers on plates inoculated with fish gut-derived *Acinetobacter*, *Plesiomonas* and *Aeromonas* strains (Fig. S4E–G). These results suggest that the reduction in the abundance of gut-derived *Acinetobacter*, *Plesiomonas* and *Aeromonas* strains by Amuc_1100 is likely mediated by the host.

The number of the *Bacillus* strain in the medium supplemented with filtered gut content supernatant from the HFD group was 1.26 × 10^9^ CFU/mL, which increased significantly to 1.76 × 10^9^ CFU/mL when the medium was supplemented with supernatant from the Amuc_1100 group (*P* < 0.05; Fig. 4E). Compared with supernatant from the HFD group, supplementation with supernatant from the AM0.01 group led to significant reductions in the abundances of *Acinetobacter* and *Aeromonas* (*P* < 0.05; Fig. 4F and H), and a non-significant downward trend in *Plesiomonas* (*P* = 0.076; Fig. 4G). These results suggest that the modulation of gut-derived *Acinetobacter*, *Plesiomonas*, and *Aeromonas* strains by Amuc_1100 may rely on certain antibacterial substances produced in the host intestine. No significant differences were observed between the AM0.01 and HFD groups in the expression of genes encoding antibacterial proteins targeting Gram-negative bacteria and antimicrobial peptides, including phospholipase A2 group III (*pla2g3*), group XIIA (*pla2g12a*), complement component 5 (*c5*), BPI fold containing family C (*bpifc*), S100 calcium binding protein A10b (*s100a10b*), hepcidin, and defensin beta-like 2 (*defbl2*) (Fig. S4H).

### Intestinal 14-3-3β/α-A is involved in the modulation of gut microbiota composition by Amuc_1100 in zebrafish

**Fig. 5:**
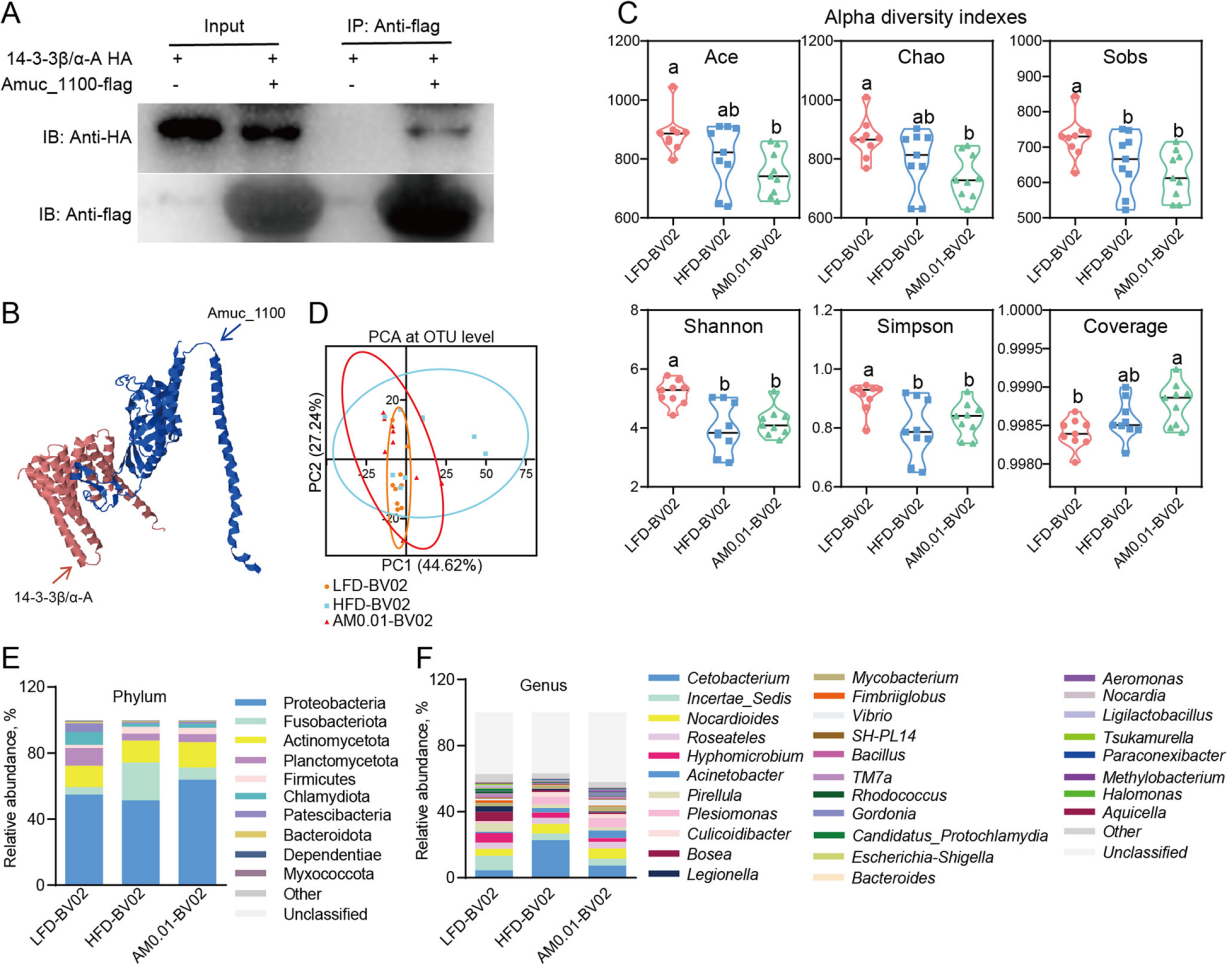
Protein 14-3-3β/α-A is involved in Amuc_1100-induced changes of gut microbiota composition. **A** Amuc_1100-flag and 14-3-3β/α-A-HA were co-expressed in HEK293T cells, and co-immunoprecipitated using anti-flag beads. Western blotting showing Amuc_1100-14-3-3β/α-A association. **B** Predicted interaction between the Amuc_1100 and 14-3-3β/α-A. **C**−**F** One-month-old zebrafish fed the BV02-supplemented LFD, HFD and AM0.01 for 4 weeks. **C** Indexes of alpha diversity (*n* = 9). **D** PCA analysis of gut microbiota at the OTU level (*n* = 9).** E** Relative abundance of gut microbial composition at the phylum level (*n* = 9). **F** Relative abundance of gut microbial composition at the genus level (*n* = 9). Data are expressed as mean ± SEM. Means without a common letter are significantly different (*P* < 0.05). Duncan's test

Pull-down assays using recombinant Amuc_1100 as bait identified 13 candidate interacting proteins by LC-MS/MS (Table 3). Among these, protein 14-3-3β/α-A was further validated by co-IP and Western blotting as an interacting partner for Amuc_1100 (Fig. 5A). Using an in vitro digestion model, Amuc_1100 was primarily digested into three fragments, including I31–K119, I43–Y137 and S138–D317 (Table S4). As predicted using DMFold and the Mol* 3D Viewer of the Protein Data Bank, the S138–D317 fragment contributed to the interaction with protein 14-3-3β/α-A. This involved two peptides (R.NERMM.P and A.QPATGAASL.T) located within the S138–D317 fragment of Amuc_1100 (Fig. 5B; Table S5).

**Table 3:** Potential interaction proteins of Amuc_1100 that obtained by pull-down assay

**Accession number**	**Protein description**	**Sublocalization**
E9QJ96	14-3-3 protein β/α-A (Fragment) OS = *Danio rerio*	Cytoplasm/Nuclear
B2GRH9	Superoxide dismutase [Cu-Zn] OS = *Danio rerio*	Cytoplasm
Q804G7	Annexin OS = *Danio rerio*	Intracellular surface of membrane
Q7SZD3	78 kDa glucose-regulated protein OS = *Danio rerio*	Endoplasmic reticulum/Nuclear
A0A2R8QH08	Fructose-bisphosphate aldolase OS = *Danio rerio*	Cytoplasm
Q3B7R7	2-phospho-D-glycerate hydro-lyase (Fragment) OS = *Danio rerio*	Cytoplasm
Q6ZM60	Calcium-transporting ATPase OS = *Danio rerio*	Membrane/Endoplasmic reticulum
Q6P967	Guanine nucleotide-binding protein (G protein), alpha-activating activity polypeptide O, b OS = *Danio rerio*	Intracellular surface of membrane
Q7ZVX2	Sodium/potassium-transporting ATPase subunit beta OS = *Danio rerio*	Membrane
Q6DGX1	Succinate-CoA ligase [ADP/GDP-forming] subunit alpha, mitochondrial OS = *Danio rerio*	Mitochondria
Z4YIG5	Small monomeric GTPase OS = *Danio rerio*	Intracellular surface of membrane
E7F1V3	TBC1 domain-containing kinase OS = *Danio rerio*	Cytoplasm/Nuclear
Q6XG62	Protein S100 OS = *Danio rerio*	Cytoplasm/Nuclear

In the absence of BV02, PCA of gut microbiota revealed partial separation among the LFD, HFD, and AM0.01 groups, with HFD and AM0.01 showing a trend toward separation along PC1 (*P* = 0.061; Fig. 3B). When 14-3-3β/α-A PPI was inhibited, the gut microbiota profile of the AM0.01-BV02 group was virtually indistinguishable from that of the HFD-BV02 group, based on alpha diversity indices and PCA (Fig. 5C and D). Upon BV02 treatment, the PCA plot among the LFD-BV02, HFD-BV02, and AM0.01-BV02 groups also showed partial separation, with HFD-BV02 and AM0.01-BV02 showing more overlap than that between no HFD and AM0.01 groups (*P* = 0.185) (Fig. 5D; Fig. S5).

In the presence of BV02, zebrafish fed the AM0.01 had a higher abundance of Proteobacteria while a lower abundance of Fusobacteriota, compared with the HFD-fed group (*P* < 0.05) (Fig. 5E; Table 4). Meanwhile, the abundance of Bacteroidota did not differ significantly between the HFD-BV02 and AM0.01-BV02 groups (Fig. 5E; Table 4). However, the differences in genus-level abundances observed between HFD and AM0.01 groups in the absence of BV02 (Table 2) were largely abolished in BV02-treated fish (Table 5). At the genus level, there was no significant difference in the abundances of *Acinetobacter* and *Plesiomonas* between the AM0.01-BV02 and HFD-BV02 groups (*P* > 0.05) (Fig. 5F; Table 5). The AM0.01-BV02 group showed a 13-fold increase in the abundance of *Aeromonas* compared to the HFD-BV02 group (*P* < 0.05) (Fig. 5F; Table 5). Meanwhile, the AM0.01-BV02 group showed no significant difference in the relative abundance of the genera *Bacillus* and *Bacteroides* compared to the HFD-BV02 group (Fig. 5F; Table 5). These results suggest that 14-3-3β/α-A plays a key role in mediating the effects of Amuc_1100 on gut microbiota composition.

**Table 4:** The relative abundance of main phyla in the intestinal microbiota of zebrafish fed the LFD-BV02, HFD-BV02 or AM0.01-BV02

Phylum	LFD-BV02	HFD-BV02	AM0.01-BV02
Proteobacteria	54.8 ± 3.13^ab^	51.4 ± 4.44^b^	63.9 ± 2.24^a^
Fusobacteriota	4.62 ± 1.49^b^	22.8 ± 7.56^a^	7.40 ± 2.89^b^
Actinomycetota	12.8 ± 1.46	13.4 ± 3.98	15.2 ± 2.41
Planctomycetota	10.8 ± 2.56^a^	4.22 ± 0.55^b^	4.97 ± 0.71^b^
Firmicutes	1.83 ± 0.51	4.09 ± 1.17	3.80 ± 1.66
Chlamydiota	7.81 ± 2.02^a^	1.84 ± 0.20^b^	2.29 ± 0.40^b^
Patescibacteria	5.33 ± 1.64^a^	0.850 ± 0.19^b^	1.30 ± 0.33^b^
Bacteroidota	1.02 ± 0.37	0.694 ± 0.28	0.424 ± 0.11
Dependentiae	0.390 ± 0.04	0.342 ± 0.07	0.271 ± 0.04
Myxococcota	0.186 ± 0.06^a^	0.0241 ± 0.01^b^	0.0793 ± 0.02^ab^

Values are expressed as the mean ± SEM (*n* = 9). Means marked with different letters differ significantly (*P* < 0.05), whereas those sharing a common letter do not. Duncan’s test

**Table 5:** The relative abundance of main genus in the intestinal microbiota of zebrafish fed the LFD-BV02, HFD-BV02 or AM0.01-BV02

Genus	LFD-BV02	HFD-BV02	AM0.01-BV02
*Cetobacterium*	4.61 ± 1.49^b^	22.8 ± 7.56^a^	7.40 ± 2.89^b^
*Incertae_Sedis*	8.67 ± 0.88^a^	4.01 ± 0.46^b^	4.22 ± 0.47^b^
*Roseateles*	3.81 ± 0.83	3.64 ± 0.88	3.86 ± 0.87
*Acinetobacter*	0.822 ± 0.25^b^	2.67 ± 0.65^ab^	4.60 ± 1.98^a^
*Pirellula*	4.90 ± 1.26^a^	2.04 ± 0.32^b^	1.73 ± 0.32^b^
*Plesiomonas*	0.843 ± 0.31^b^	4.86 ± 1.41^ab^	5.69 ± 1.97^a^
*Culicoidibacter*	0.513 ± 0.24	2.89 ± 1.19	2.48 ± 1.75
*Bosea*	5.69a ± 2.04	0.912b ± 0.13	0.929b ± 0.18
*Legionella*	3.38a ± 0.43	0.968b ± 0.24	0.686b ± 0.19
*Fimbriiglobus*	1.48a ± 0.42	0.317b ± 0.04	0.459b ± 0.11
*SH-PL14*	0.673 ± 0.10	0.513 ± 0.10	0.487 ± 0.10
*Bacillus*	0.488 ± 0.28	0.325 ± 0.14	0.777 ± 0.25
*TM7a*	2.09 ± 0.66^a^	0.350 ± 0.09^b^	0.655 ± 0.17^b^
*Gordonia*	0.481 ± 0.09^ab^	0.305 ± 0.13^b^	1.69 ± 0.71^a^
*Candidatus_Protochlamydia*	1.18 ± 0.49^a^	0.123 ± 0.02^b^	0.107 ± 0.04^b^
*Bacteroides*	0.216 ± 0.09	0.129 ± 0.05	0.0678 ± 0.01
*Aeromonas*	0.0893 ± 0.05^b^	0.135 ± 0.06^b^	1.26 ± 0.62^a^
*Nocardia*	1.36 ± 0.58^a^	0.171 ± 0.06^b^	0.254 ± 0.11^b^
*Ligilactobacillus*	0.209 ± 0.08	0.206 ± 0.08	0.0644 ± 0.02
*Methylobacterium*	0.313 ± 0.09	0.111 ± 0.02	0.195 ± 0.08
*Halomonas*	0.235 ± 0.06	0.194 ± 0.04	0.166 ± 0.03

Values are expressed as the mean ± SEM (*n* = 9). Means marked with different letters differ significantly (*P* < 0.05), whereas those sharing a common letter do not. Duncan’s test

### Intestinal 14-3-3β/α-A mediates the alleviation of hepatic lipid accumulation by Amuc_1100

**Fig. 6:**
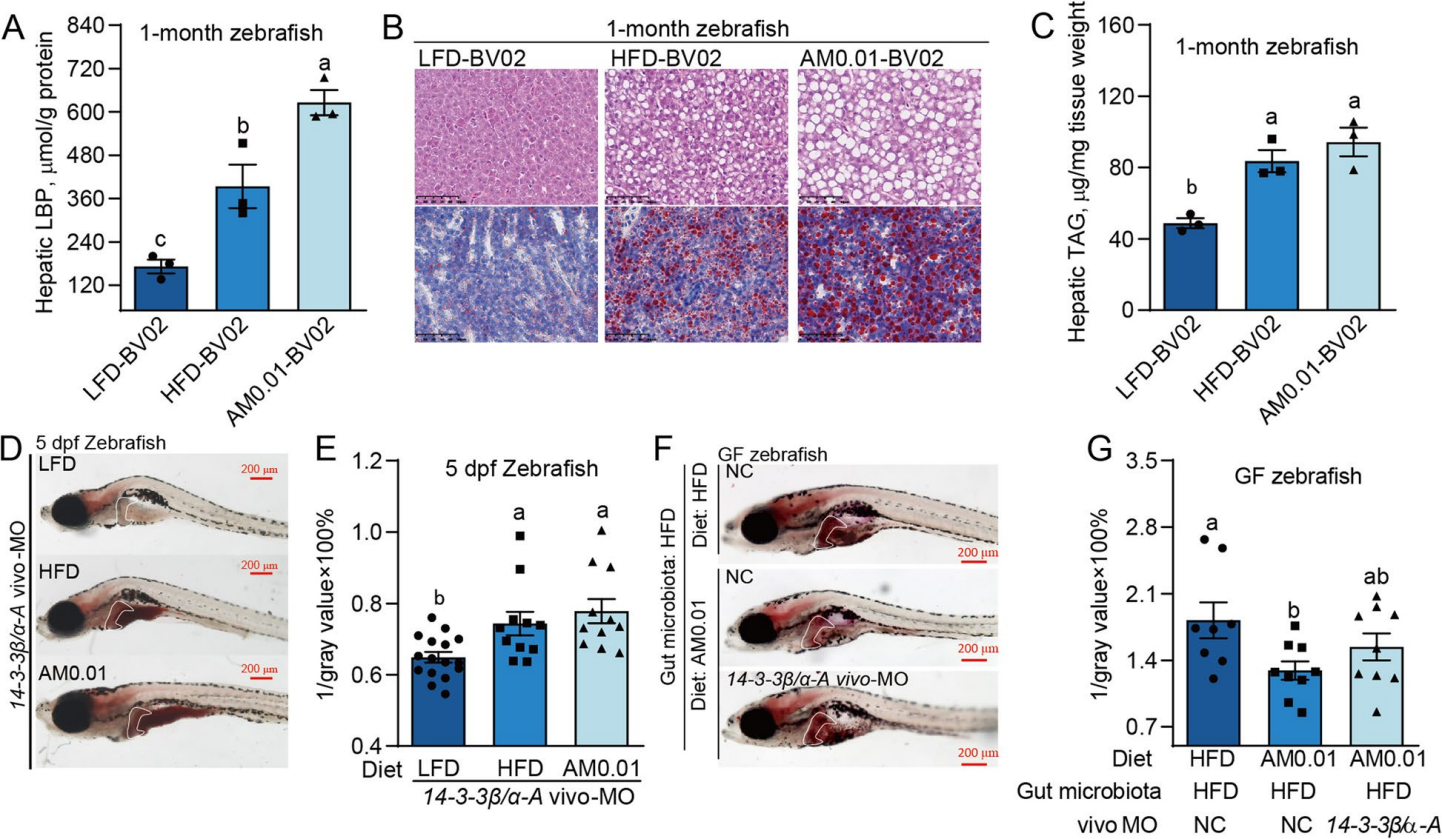
Intestinal 14-3-3β/α-A is indispensable to the alleviation of Amuc_1100 to hepatic lipid accumulation. **A**−**C** One-month-old zebrafish fed on BV02-supplemented LFD, HFD and AM0.01 for 4 weeks. **A** Hepatic LBP levels (*n* = 3). **B** Representative images of H&E- and ORO-stained liver sections. The scale bar = 50 μm. **C** Hepatic TAG contents (*n* = 3). **D** and **E** Zebrafish at 5 dpf were transfected with vivo-MO targeting the *14-3-3β/α-A* and fed on the LFD, HFD or AM0.01 for 7 d. **D** Representative images of whole-mount ORO staining. The scale bar = 200 μm. **E** Quantitative assessment of whole-mount ORO staining (*n* = 11 or 16). **F**−**G** GF zebrafish at 5 dpf were transferred with the HFD-gut microbiota and fed the HFD or AM0.01. Then AM0.01-fed fish were transfected with std-MO (NC) or *14-3-3β/α-A*-targeted vivo-MO. **F** Representative images of whole-mount ORO staining. The scale bar = 200 μm. **G** Quantitative assessment of whole-mount ORO staining (*n* = 8 or 9). Data are expressed as mean ± SEM. Means without a common letter are significantly different (*P* < 0.05). Duncan’s test

Compared with the HFD-BV02 group, the AM0.01-BV02 group exhibited a higher hepatic LBP level (*P* < 0.05; Fig. 6A). Meanwhile, the area of large intracellular lipid droplets and the hepatic TAG level in the AM0.01-BV02 group were comparable to those in the HFD-BV02 group (*P* > 0.05; Fig. 6B and C). In 5-dpf zebrafish larvae with *14-3-3β/α-A* knockdown, the red staining intensity and mean gray value in the liver area did not differ between the AM0.01 and HFD groups (*P* > 0.05; Fig. 6D and E). The efficiency of *14-3-3β/α-A* vivo-MO is presented in Fig. S6A. These results suggest that protein 14-3-3β/α-A may partly mediate the alleviation of hepatic lipid accumulation by Amuc_1100. Compared to HFD-fed GF zebrafish transplanted with HFD-BV02 microbiota, those receiving AM0.01-BV02 microbiota showed no significant difference in intrahepatic red staining or mean gray values (Fig. S6B and C). These results demonstrate that 14-3-3β/α-A plays an essential role in enabling the gut microbiota to reduce hepatic lipid accumulation.

To further investigate the role of protein 14-3-3β/α-A in the alleviation of hepatic lipid accumulation by Amuc_1100, GF zebrafish receiving HFD gut microbiota were fed either the HFD or the AM0.01. Compared to those fed the HFD, zebrafish larvae colonized with HFD gut microbiota and fed the AM0.01 showed reduced intrahepatic red staining and lower mean gray values (*P* < 0.05; Fig. 6F and G). However, knockdown of *14-3-3β/α-A* exacerbated hepatic lipid deposition, as indicated by increased staining intensity and mean gray value in the liver of HFD microbiota-colonized larvae fed the AM0.01 (Fig. 6F and G).

## Discussion

The use of HFDs is common in aquaculture to enhance production efficiency and reduce feed costs. However, this practice often leads to an overload of FFAs, which contributes to hepatic lipid accumulation and metabolic disorders in fish [3–5]. This study demonstrates that intestinal 14-3-3β/α-A interacts with Amuc_1100, resulting in a beneficial remodeling of the gut microbiota that ameliorates hepatic lipid accumulation. Furthermore, we identify 14-3-3β/α-A as an intracellular PRR for Amuc_1100, underscoring its essential role in mediating the gut microbiota-dependent alleviation of hepatic steatosis. These findings establish a foundation for developing Amuc_1100-based strategies to counteract HFD-induced hepatic lipid accumulation in aquaculture.

In this study, the HFD containing 16% fat effectively induced hepatic lipid accumulation, which is consistent with other established zebrafish models [50, 51]. Amuc_1100 supplementation significantly alleviated HFD-induced hepatic lipid accumulation in zebrafish, as evidenced by reduced hepatic TAG content, attenuated histological steatosis, and lower serum Alt levels. These phenotypic improvements were accompanied by distinct changes in the expression of genes regulating hepatic lipid metabolism. Hepatic lipid accumulation results from an imbalance between lipid synthesis (de novo lipogenesis, DNL) and lipid catabolism (fatty acid oxidation) [52]. Amuc_1100 administration downregulated key lipogenic genes, including *srebf1*, *acc1*, *fas*, and *c/ebpα*. Mammalian SREBP1 is a master transcription factor that activates the expression of lipogenic enzymes such as ACC1 and FAS, driving DNL [53]. C/EBPα, which is positively correlated with hepatic steatosis severity, can further potentiate lipogenic gene expression through cooperation with SREBP1 [54]. The coordinated downregulation of these genes suggests that Amuc_1100 suppresses endogenous fatty acid synthesis, contributing to reduced lipid accumulation. Conversely, Amuc_1100 feeding upregulated genes involved in lipid catabolism, including *atgl* and *cpt1aa*. ATGL catalyzes the rate-limiting step of TAG lipolysis, releasing fatty acids for subsequent β-oxidation [55, 56]. CPT1 is the key enzyme controlling mitochondrial fatty acid import and oxidation [57]. The upregulation of these genes indicates that Amuc_1100 promotes fatty acid utilization, further counteracting HFD-induced lipid overload. Notably, the effects of Amuc_1100 on hepatic lipid metabolism appear to be indirect, as Amuc_1100 failed to reduce TAG accumulation in ZFL cells exposed to OA and had no effect in GF zebrafish. These findings indicate that Amuc_1100 does not act directly on hepatocytes, but rather requires the presence of gut microbiota to exert its hepatoprotective effects. This interpretation is consistent with our subsequent experiments demonstrating that the gut microbiota is both necessary and sufficient for Amuc_1100-mediated alleviation of hepatic steatosis.

This study observed reductions in serum endotoxin and hepatic LBP, two bactericidal permeability-increasing indicators [58]. It suggests an improvement in gut-liver axis homeostasis by Amuc_1100 in zebrafish. As a key component of the gut-liver axis, the gut microbiota is implicated in the pathogenesis of hepatic steatosis [59]. And a healthy gut microbiota plays a vital role in maintaining liver homeostasis [60]. In this study, the genera *Acinetobacter*, *Plesiomonas* and *Aeromonas* were enriched in conventional HFD-fed zebrafish. The genus *Acinetobacter* is positively associated with HFD-induced hepatic steatosis in rats [61–63]. A reduction in *Acinetobacter* abundance underlies the alleviation of hepatic steatosis by prebiotics like inulin and isomalt in rats [61, 62]. Furthermore, gut microbiota dysbiosis marked by an enrichment of the genus *Acinetobacter* contributes to diet-induced hepatic steatosis in GF mice [64]. Genera *Plesiomonas* and *Aeromonas* are positively correlated with lipid synthesis in the zebrafish liver [65, 66]. According to these reports, HFD feeding resulted in a gut microbiota profile promoting hepatic lipid accumulation in zebrafish.

A critical question is whether the Amuc_1100-induced shift in gut microbiota is a causal driver of reduced hepatic lipid accumulation or merely a correlative epiphenomenon. Our study provides strong evidence for causality through several lines of inquiry. The pivotal finding comes from our GF zebrafish transplant experiment. GF zebrafish colonized with microbiota derived from AM0.01-fed donors recapitulated the protective hepatic phenotype, exhibiting significantly less lipid accumulation than those colonized with HFD-derived microbiota. These results unequivocally demonstrate that the altered microbial community itself is sufficient to transmit the beneficial effect, independent of direct host exposure to Amuc_1100.

Furthermore, the specific taxonomic changes we observed are consistent with established causal roles of these bacteria in liver health. The enrichment of *Bacillus* and *Bacteroides* in AM0.01 group aligns with numerous studies where supplementation with these genera directly ameliorates diet-induced hepatic steatosis through the production of bioactive metabolites like short-chain fatty acids and L-arginine in both mice and zebrafish [67–73]. Conversely, the depletion of *Acinetobacter*, *Plesiomonas*, and *Aeromonas* is equally significant. These Gram-negative genera are potent producers of LPS, and their increased abundance has been causally linked to hepatic inflammation and steatosis by promoting endotoxin translocation and triggering inflammatory cascades that exacerbate lipid dysregulation [61–64, 74, 75]. Therefore, the observed shift is not a random fluctuation but a directed restructuring of the microbiota towards a well-documented hepatoprotective profile.

In this study, PERMANOVA revealed that while both HFD and AM0.01 groups differed significantly from the LFD group (*P* < 0.05), the difference between HFD and AM0.01 groups did not reach statistical significance (*P* = 0.061). This pattern explains the considerable overlap observed in the PCA plot and reflects the high within-group variation inherent to gut microbiota studies [76–79]. Potential sources of this high within-group variation include individual biological variation (stochastic colonization, genetic heterogeneity), technical factors (sequencing depth, DNA extraction efficiency), and the inherent instability of HFD-induced dysbiosis [80]. Importantly, the lack of statistical significance at the community level does not negate the biological relevance of the Amuc_1100-induced changes as revealed by statistically significant changes of these specific taxa with established roles in lipid metabolism (*Bacillus*, *Bacteroides*, *Acinetobacter*, *Plesiomonas*, and *Aeromonas*), and significantly reduced hepatic lipid accumulation in GF zebrafish colonized with AM0.01-derived microbiota. These evidence demonstrates that the community-level differences, although subtle in overall structure, are sufficient to transmit a phenotypic effect.

In this study, the inhibition of fish gut-derived *Acinetobacter*, *Plesiomonas* and *Aeromonas* strains by Amuc_1100 occurred in vivo but not ex vivo, indicating that its regulatory effect is mediated by the host, at least for these three strains. However, a role for antimicrobial peptides, which play a part in host selective immunity and barrier defense, was ruled out in this study due to the lack of a significant difference in their intestinal expression levels between the HFD and AM0.01 groups. This finding is consistent with a previous study where Amuc_1100 also failed to reverse the HFD-induced suppression of antimicrobial peptides, including lysozyme 1, α-defensin, regenerating family member 3 gamma, and phospholipase A2 group-II, in mice [20].

Amuc_1100 has been identified as a ligand of human TLR2 [19, 20]. However, no interaction between the zebrafish Tlr2 and Amuc_1100 was observed in this study. This lack of interaction is likely attributable to the ligand specificity of fish Tlr2 [81]. We identified the cytosolic protein 14-3-3β/α-A as the interacting partner in this study. This protein is an isoform of the 14-3-3 family, a series of highly conserved proteins found in all eukaryotic organisms [82, 83]. Localized to multiple subcellular sites including the plasma membrane, endoplasmic reticulum, Golgi apparatus, nucleus, microtubules, mitochondria, and cytoskeletal fibers, 14-3-3 proteins extensively regulate pathways for signal transduction, checkpoint control, apoptosis, and nutrient-sensing [82, 84]. It has been demonstrated that 14-3-3β/α-A acts as an intracellular PRR in zebrafish, which can identify various microbial molecules including peptidoglycan, lipoteichoic acid, and LPS [33]. This protein also exhibits antimicrobial effector functions by depolarizing the membranes of both Gram-negative and Gram-positive bacteria [33]. These reports collectively indicate that 14-3-3β/α-A is involved in coordinating host-microbe interactions [33, 34].

To validate the functional role of 14-3-3β/α-A, we employed both pharmacological inhibition with BV02 and gene-specific knockdown with vivo-MO. BV02 is a reference inhibitor of 14-3-3 PPI, with direct binding to 14-3-3 proteins confirmed by NMR spectroscopy [39] and functional validation through disruption of 14-3-3/client interactions [40]. Molecular docking studies indicate that BV02 binds to multiple 14-3-3 family members [85], consistent with its role as a pan-14-3-3 inhibitor. Importantly, the concordance between BV02 treatment and 14-3-3β/α-A-specific knockdown provides orthogonal validation that the observed phenotypes are specifically attributable to inhibition of this isoform.

Our study establishes the indispensable role of 14-3-3β/α-A as an intracellular receptor for Amuc_1100 in mediating microbiota modulation. In this study, Amuc_1100 did not reduce the abundance of *Acinetobacter*, *Plesiomonas* and *Aeromonas* upon inhibition of 14-3-3β/α-A PPI, indicating that its modulation of the gut microbiota is partly dependent on its interaction with 14-3-3β/α-A. However, the precise downstream mechanisms by which this interaction reshapes microbial communities warrant further investigation.

14-3-3 proteins are known to regulate the activity of plasma membrane H^+^-ATPases in plants, thereby controlling extracellular pH [86]. Given the evolutionary conservation of 14-3-3 proteins, we hypothesize that in zebrafish, this interaction might similarly stimulate proton pumping into the intestinal lumen. Previous studies have shown that *Acinetobacter baumannii* exhibits reduced growth and biofilm formation at pH 4.5, while *Bacillus* spores maintain viability even under acidic conditions [87, 88]. *Aeromonas* species also display sensitivity to acidic pH, with cell death occurring when pH drops below 6.5 [89, 90]. This differential sensitivity to pH is consistent with our hypothesis that Amuc_1100-induced 14-3-3β/α-A activation may acidify the intestinal lumen, thereby selectively suppressing *Acinetobacter* and *Aeromonas* while favoring *Bacillus* colonization. The resulting local acidification could create an unfavorable environment for pH-sensitive, potentially genera like *Acinetobacter* and *Aeromonas*, while having a neutral effect on *Bacillus*. This hypothesis is consistent with our observation that Amuc_1100 administration did not alter the expression of classic host antimicrobial peptides, suggesting a mechanism independent of the canonical immune response. Alternatively, the direct antimicrobial effector function recently attributed to zebrafish 14-3-3β/α-A [85] could be potentiated upon Amuc_1100 binding. Future studies should focus on directly measuring intestinal pH and characterizing the specific antimicrobial activity of the 14-3-3β/α-A-Amuc_1100 complex to validate these proposed mechanisms.

The growth of the fish gut-derived *Bacillus* strain was found to be stimulated by either Amuc_1100 or its corresponding gut content supernatant in an ex vivo system. Given that 14-3-3β/α-A is reported to exert antimicrobial activity against *Bacillus* [33], the observed enrichment of the genus *Bacillus* in the gut of Amuc_1100-fed zebrafish is likely independent of 14-3-3β/α-A. *Bacillus* may utilize Amuc_1100 as a nitrogen source. Additionally, mammalian hosts can metabolize Amuc_1100 into nutritive substances such as arginine, which can nourish beneficial gut microbes, or into neurotransmitters that indirectly influence bacterial colonization [72, 91, 92]. The digestive products of Amuc_1100 in the intestinal lumen may include bioactive compounds that stimulate the growth of the genus *Bacillus*, analogous to its documented effect on L-arginine-producing *Bifidobacterium* species [72]. Additionally, competition among *Bacillus*, *Acinetobacter* and *Aeromonas* has been documented [93, 94]. This competitive dynamic suggests that the enrichment of *Bacillus* may be partly due to the expansion of its ecological niche following the diminishment of *Acinetobacter*, *Plesiomonas* and *Aeromonas*.

**Fig. 7:**
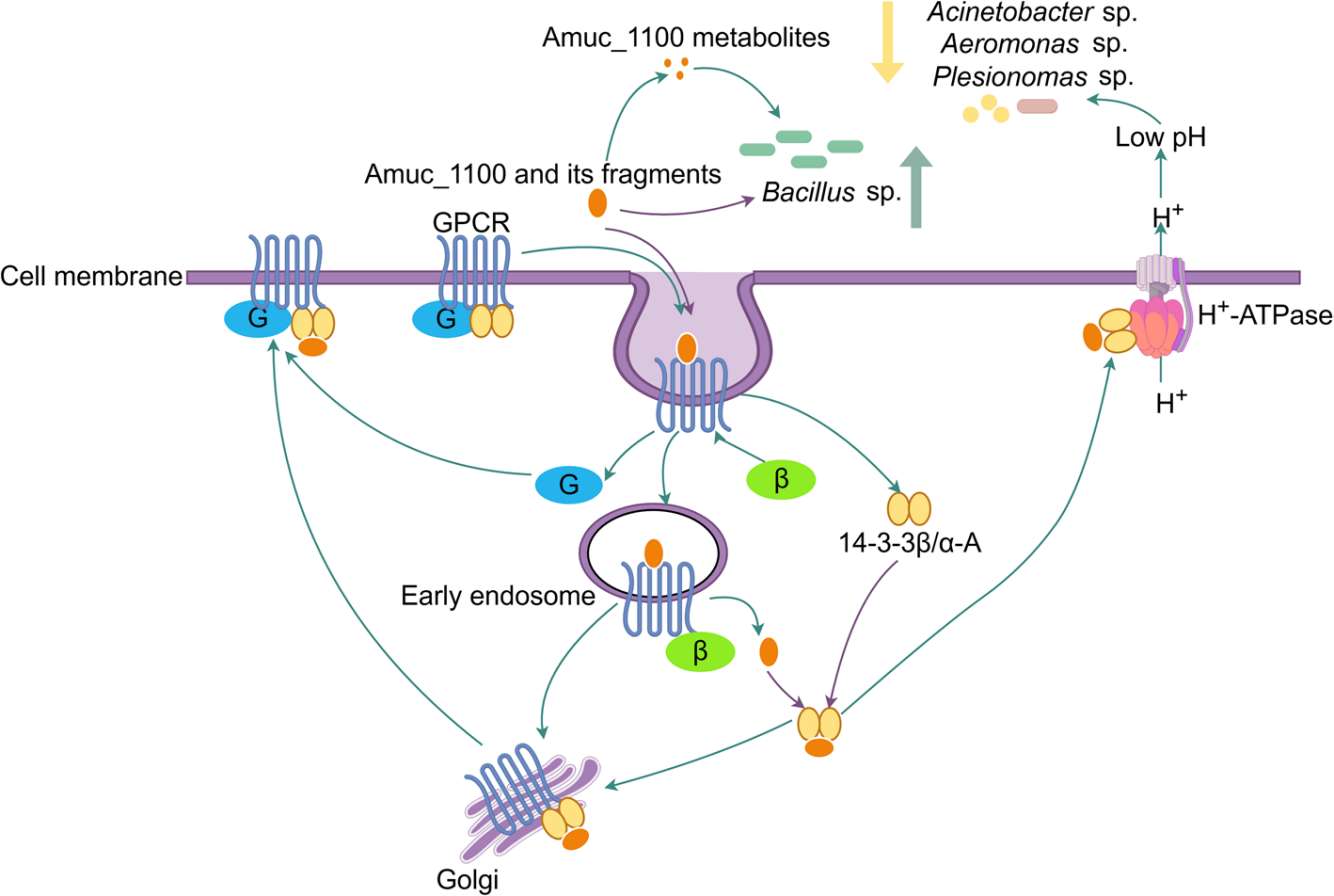
A proposed model illustrating the mechanism by which Amuc_1100 enters the cytoplasm via GPCR-mediated endocytosis, interacts with 14-3-3β/α-A, and subsequently modulates gut microbiota, based on our results and literature evidence. Released Amuc_1100 gets approach and interacts to 14-3-3β/α-A. Then, Amuc_1100-binded 14-3-3β/α-A may further activate the membrane located H^+^-ATPase and coordinate H^+^ pumping into the intestinal lumen, leading to the low pH in the lumen. Low pH in the intestinal lumen may produce anti-bacterial effect to *Acinetobacter*, *Aeromonas* and *Plesiomonas*. Meanwhile, *Bacillus* may be promoted by directly utilizing Amuc_1100 or its digestive products as a nitrogen source. Green line: the inference made based on the literature evidence; violet line: findings in this study. G, G proteins; GPCR, G protein coupled receptor; β, β-arrestins. This figure is produced by Figdraw

Fish 14-3-3β/α-A is primarily localized to the cytoplasm [33, 34], raising the question of how Amuc_1100 gains access to its intracellular interactor. Beyond their presence in the cytoplasm, organelles, and nucleus, 14-3-3 proteins facilitate receptor trafficking and receptor-mediated endocytosis by binding to membrane proteins as essential accessory factors [95]. The cell leverages the pH-dependent dissociation between receptors and ligands. This rapid dissociation in the acidic environment of early endosomes enables receptors to recycle to the plasma membrane and promotes the release of ligands into the cytosol [96]. G protein coupled receptor-mediated endocytosis is a transport pathway of macromolecular substances in the intestine [97]. Our in vitro uptake experiment showed that Amuc_1100-GFP can enter ZF4 cells and accumulate in the perinuclear region (Fig. S7), demonstrating that Amuc_1100 can cross the plasma membrane, at least in cultured cells. Simulated intestinal digestion generated three fragments of Amuc_1100, one of which (S138–D317) contains the predicted 14-3-3 interaction motifs (R.NERMM.P and A.QPATGAASL.T). Evidence indicates that 14-3-3β/α-A facilitates the endocytic process, enabling viral entry into FHM cells [34]. Based on these observations and published evidence that 14-3-3 proteins can facilitate endocytosis [34, 94], we propose that receptor-mediated endocytosis may be involved in Amuc_1100 internalization (Fig. 7). However, the exact internalization pathway and whether the full-length protein or its fragments mediate the biological effects require further investigation.

Given the evolutionary conservation of both 14-3-3 proteins and lipid metabolism pathways between zebrafish and mammals, these findings may also have implications for understanding host-microbiota interactions in the context of human NAFLD and other metabolic disorders. The identification of 14-3-3β/α-A as a host factor mediating microbiota remodeling by a bacterial protein highlights a potential target for therapeutic strategies aimed at restoring gut-liver axis homeostasis.

Although we identified 14-3-3β/α-A as an essential host factor for Amuc_1100-induced gut microbiota remodeling, the precise downstream molecular events following this interaction remain to be elucidated. Besides, this study did not evaluate immune responses or potential allergic reactions associated with Amuc_1100 administration. While Amuc_1100 is derived from *A. muciniphila*, a bacterium with context-dependent effects on type 2 immunity [15, 16], our study focused on the mechanism of action rather than safety assessment. Future studies should directly assess whether Amuc_1100 influences type 2 immune responses or allergic outcomes, particularly in the context of long-term administration. Future studies should address these limitations to further validate and extend our findings.

## Conclusions

This study revealed that Amuc_1100 alleviates hepatic lipid accumulation by modulating the gut microbiota, a process mediated by intestinal 14-3-3β/α-A. Amuc_1100 restricts the growth of microbes such as *Acinetobacter*, *Plesiomonas* and *Aeromonas*, while promoting the growth of *Bacillus* in the gut in a 14-3-3β/α-A-dependent manner. A balanced gut microbiota plays a role in alleviating HFD-induced hepatic lipid accumulation in zebrafish. This study elucidates the role of gut microbiota in the Amuc_1100-mediated alleviation of hepatic lipid accumulation and confirms that Amuc_1100 is a potential active component for novel therapeutic strategies derived from *A. muciniphila*. The role of 14-3-3β/α-A in reshaping gut microbiota composition highlights its potential as a therapeutic target for strategies aimed at maintaining microbiota homeostasis and reducing hepatic lipid accumulation.

## Supplementary Information

Additional file 1: Fig. S1 Effects of Amuc_1100 on the growth performance and hepatic TAG of 1-month-old zebrafish, and intracellular TAG accumulation in ZFL cells. Fig. S2 Effects of Amuc_1100 on hepatic steatosis in GF zebrafish. Fig. S3 PERMANOVA at the OTU level among the LFD, HFD, and AM0.01 groups after a 4-week feeding period. Fig. S4 Effects of Amuc_1100 on fish gut-derived strains ex vivo. Fig. S5 PERMANOVA at the OTU level among the LFD-BV02, HFD-BV02, and AM0.01-BV02 groups after a 4-week feeding period. Fig. S6 Effects of LFD-BV02, HFD-BV02 or AM0.01-BV02 gut microbiota on HFD-induced hepatic steatosis in GF zebrafish. Fig. S7 Representative images showing the intracellular location of exogenous Amuc_1100-GFP in ZF4 cells. Table S1 Ingredients for 1-month-old zebrafish diet (g/kg dry diet). Table S2 Ingredients for zebrafish larvae diet at 5 dpf (g/kg dry diet). Table S3 Sequences of primers. Table S4 Fragments that identified by mass spectrometry after digestion in vitro. Table S5 Predicted amino acid residues involving the interaction between the Amuc_1100 and 14-3-3β/α-A.

Additional file 2. Original Western blot images.

## Data Availability

The datasets used during the current study are available from the corresponding author on reasonable request.

## Abbreviations

ABS: Antibiotics
*acc1*: Acetyl-CoA carboxylase 1
Alt: Alanine transaminase
*atgl*: Adipose triglyceride lipase
*c/ebpα*: CCAAT/enhancer-binding protein alpha
CFU: Colony forming units
co-IP: Co-immunoprecipitation
*cpt1aa*: Carnitine palmitoyltransferase 1Aa
*dgat*: Diacylglycerol acyltransferase
DNL: De novo lipogenesis
dpf: Days post fertilization
ER: Endoplasmic reticulum
*fas*: Fatty acid synthase
FFAs: Free fatty acids
GF: Germ-free
GZM: Gnotobiotic zebrafish medium
H&E: Hematoxylin and eosin
AM0.01: HFD supplemented with 0.01% Amuc_1100
HFD: High-fat diet
LB: Lysogeny broth
LBP: Lipopolysaccharide-binding protein
LC-MS: Liquid chromatography-mass spectrometry
L-D: Light-dark
LFD: Low-fat diet
LPS: Lipopolysaccharides
MO: Morpholino oligonucleotides
MS222: Tricaine methanesulfonate
NAFLD: Non-alcoholic fatty liver disease
OA: Oleic acid
OD: Optical density
ORO: Oil Red O
OTU: Operational taxonomic units
PCA: Principal component analysis
*pparγ*: Peroxisome proliferator-activated receptor gamma
PPI: Protein-protein interaction
PRR: Pattern recognition receptor
PVDF: Polyvinylidene difluoride
SDS: Sodium dodecyl sulphate
*sreb**f1*: Sterol regulatory element binding transcription factor 1
TAG: Triacylglycerol
TLR2: Toll-like receptor 2
ZFL: Zebrafish liver cells

## References

[1] Fishery Bureau of Agriculture Ministry of China. Chinese fishery statistical yearbook. Beijing (in Chinese): China Agriculture Press; 2025.

[2] The Ministry of Agriculture and Rural Affairs of the People’s Republic of China. China agricultural outlook report (2025–2034). (in Chinese). 2025. https://scs.moa.gov.cn/gzdt/202504/t20250424_6473502.htm.

[3] Xu J, Li XY, Yao XZ, Xie SW, Chi SY, Zhang S, et al. Protective effects of bile acids against hepatic lipid accumulation in hybrid grouper fed a high-lipid diet. Front Nutr. 2022;9:813249. 10.3389/fnut.2022.813249. 35145986 10.3389/fnut.2022.813249 PMC8821168

[4] Zheng H, Xu YC, Zhao T, Luo Z, Zhang DG, Song CC, et al. Dietary chenodeoxycholic acid attenuates high-fat diet-induced growth retardation, lipid accumulation and bile acid metabolism disorder in the liver of yellow catfish *Pelteobagrus fulvidraco*. Br J Nutr. 2024;131(6):921–34. 10.1017/S0007114523002489. 37905695 10.1017/S0007114523002489

[5] Jia R, Hou YR, Zhang LQ, Li B, Zhu J. Effects of berberine on lipid metabolism, antioxidant status, and immune response in liver of tilapia (*Oreochromis niloticus*) under a high-fat diet feeding. Antioxidants. 2024;13(5):548. 10.3390/antiox13050548. 38790653 10.3390/antiox13050548 PMC11117941

[6] Gao J, Mang Q, Sun Y, Xu GC. Probiotic supplementation improves lipid metabolism disorders and immune suppression induced by high-fat diets in *Coilia nasus* liver. Biology. 2025;14(4):381. 10.3390/biology14040381. 40282246 10.3390/biology14040381 PMC12024547

[7] Zhou W, Xie MX, Xie YD, Liang H, Li M, Ran C, et al. The effect of dietary supplementation of *Lactobacillus rhamnosus* GCC-3 fermentation product on gut and liver health, and resistance against bacterial infection of the genetically improved farmed tilapia (GIFT, *Oreochromis niloticus*). Aquaculture. 2022;558:738326. 10.1016/j.aquaculture.2022.738326. 10.1016/j.fsi.2022.04.019 35430347

[8] Zhang BY, Yu HX, Yang HL, Cai GH, Sun YZ. Paraprobiotic and postbiotic forms of *Bacillus altitudinis* F7 relieved high starch diet-induced growth retardation, glycolipid metabolism disorders and liver damage in *Micropterus salmoides*. Aquac Rep. 2025;45:103249. 10.1016/j.aqrep.2025.103249.

[9] Li M, Liang H, Zhang J, Chen J, Xu S, Zhou W, et al. *Bacillus subtilis* HGCC-1 improves growth performance and liver health via regulating gut microbiota in golden pompano. Anim Microbiome. 2025;7:7. 10.1186/s42523-024-00372-x. 39806437 10.1186/s42523-024-00372-x PMC11731533

[10] Derrien M, Vaughan EE, Plugge CM, de Vos WM. *Akkermansia muciniphila* gen. nov., sp nov., a human intestinal mucin-degrading bacterium. Int J Syst Evol Micr. 2004;5(54):1469–76. 10.1099/ijs.0.02873-0. 10.1099/ijs.0.02873-0 15388697

[11] Ouwerkerk JP, van der Ark KCH, Davids M, Claassens NJ, Finestra TR, de Vos WM, et al. Adaptation of *Akkermansia muciniphila* to the oxic-anoxic interface of the mucus layer. Appl Environ Microbiol. 2016;82(23):6983–93. 10.1128/AEM.01641-16. 27663027 10.1128/AEM.01641-16 PMC5103097

[12] Cani PD, Depommier C, Derrien M, Everard A, de Vos WM. *Akkermansia muciniphila*: paradigm for next-generation beneficial microorganisms. Nat Rev Gastroenterol Hepatol. 2022;19:625–37. 10.1038/s41575-022-00631-9. 35641786 10.1038/s41575-022-00631-9

[13] Li LF, Li MC, Chen YH, Yu ZY, Cheng P, Yu ZD, et al. Function and therapeutic prospects of next-generation probiotic *Akkermansia muciniphila* in infectious diseases. Front Microbiol. 2024;15:1354447. 10.3389/fmicb.2024.1354447. 38384263 10.3389/fmicb.2024.1354447 PMC10880487

[14] Lei WH, Cheng YW, Gao J, Liu X, Shao L, Kong QM, et al. *Akkermansia muciniphila* in neuropsychiatric disorders: friend or foe? Front Cell Infect Microbiol. 2023;13:1224155. 10.3389/fcimb.2023.1224155. 37492530 10.3389/fcimb.2023.1224155 PMC10363720

[15] Sugihara K, Kitamoto S, Saraithong P, Nagao-Kitamoto H, Hoostal M, McCarthy C, et al. Mucolytic bacteria license pathobionts to acquire host-derived nutrients during dietary nutrient restriction. Cell Rep. 2022;40(3):111093. 10.1016/j.celrep.2022.111093. 35858565 10.1016/j.celrep.2022.111093 PMC10903618

[16] Parrish A, Boudaud M, Grant ET, Willieme S, Neumann M, Wolter M, et al. *Akkermansia muciniphila* exacerbates food allergy in fibre-deprived mice. Nat Microbiol. 2023;8(10):1863–79. 10.1038/s41564-023-01464-1. 37696941 10.1038/s41564-023-01464-1 PMC10522492

[17] Faghfuri E, Gholizadeh P. The role of *Akkermansia muciniphila* in colorectal cancer: A double-edged sword of treatment or disease progression? Biomed Pharmacother. 2024;173:116416. 10.1016/j.biopha.2024.116416. 38471272 10.1016/j.biopha.2024.116416

[18] Zhang W, Xi G, Zhang H, Bi J, Zhou T, Zhu J, et al. Pasteurized *Akkermansia muciniphila* promotes GP2 expression in microfold cells and facilitates *Salmonella* infection. Protein Cell. 2025;16(9):829–34. 10.1093/procel/pwaf017. 10.1093/procel/pwaf017 40037364

[19] Wang JC, Xiang R, Wang RJ, Zhang BC, Gong WM, Zhang JC, et al. The variable oligomeric state of Amuc_1100 from *Akkermansia muciniphila*. J Struct Biol. 2020;212(1):107593. 10.1016/j.jsb.2020.107593. 32736072 10.1016/j.jsb.2020.107593

[20] Plovier H, Everard A, Druart C, Depommier C, Van Hul M, Geurts L, et al. A purified membrane protein from *Akkermansia muciniphila* or the pasteurized bacterium improves metabolism in obese and diabetic mice. Nat Med. 2017;23:107–13. 10.1038/nm.4236. 27892954 10.1038/nm.4236

[21] Qu DN, Chen MY, Zhu HY, Liu XY, Cui YA, Zhou W, et al. *Akkermansia muciniphila* and its outer membrane protein Amuc_1100 prevent high-fat diet-induced nonalcoholic fatty liver disease in mice. Biochem Biophys Res Commun. 2023;684:149131. 10.1016/j.bbrc.2023.149131. 37866242 10.1016/j.bbrc.2023.149131

[22] Cheng JY, Lei ZY, Fang C, Jia W, Xu Y. Pasteurized *Akkermansia muciniphila* and its outer membrane protein Amuc_1100 alleviate alcoholic liver disease through modulating gut microbiota and host metabolism. Food Biosci. 2024;59:104072. 10.1016/j.fbio.2024.104072.

[23] Kim S, Lee Y, Kim Y, Seo Y, Lee H, Ha J, et al. *Akkermansia muciniphila* prevents fatty liver disease, decreases serum triglycerides, and maintains gut homeostasis. Appl Environ Microbiol. 2020;86(7):e03004-19. 10.1128/AEM.03004-19. 31953338 10.1128/AEM.03004-19 PMC7082569

[24] Zhang FL, Yang YL, Zhang Z, Yao YY, Xia R, Gao CC, et al. Surface-Displayed Amuc_1100 from *Akkermansia muciniphila* on ZHY1 improves hepatic steatosis and intestinal health in high-fat-fed zebrafish. Front Nutr. 2021;8:726108. 10.3389/fnut.2021.726108. 34722607 10.3389/fnut.2021.726108 PMC8548614

[25] Yang GK, Yin MY, Guo SH, Yang BY, Gu JN, Zhang Y, et al. Recombinant Amuc_1100 *Lactococcus lactis* regulates metabolism and intestinal health of largemouth bass fed high starch diet. Aquaculture. 2026;612:743232. 10.1016/j.aquaculture.2025.743232.

[26] Dahm R, Geisler R. Learning from small fry: the zebrafish as a genetic model organism for aquaculture fish species. Mar Biotechnol. 2006;8(4):329–45. 10.1007/s10126-006-5139-0. 10.1007/s10126-006-5139-0 16670967

[27] Awad K, Huang J, Rajashekar D, Duque G, Brotto M, Karasik D. Zebrafish as a model for lipidomics and similar investigations. Methods Mol Biol. 2024;2816:13–24. 10.1007/978-1-0716-3902-3_2. 38977584 10.1007/978-1-0716-3902-3_2

[28] Quinlivan Vanessa H, Farber SA. Lipid uptake, metabolism, and transport in the larval zebrafish. Front Endocrinol. 2017;8:319. 10.3389/fendo.2017.00319. 10.3389/fendo.2017.00319 PMC5701920 29209275

[29] Chang C, Li HC, Zhang RL. Zebrafish facilitate non-alcoholic fatty liver disease research: tools, models and applications. Liver Int. 2023;43(7):1385–98. 10.1111/liv.15601. 37122203 10.1111/liv.15601

[30] Pham LN, Kanther M, Semova I, Rawls JF. Methods for generating and colonizing gnotobiotic zebrafish. Nat Protoc. 2008;3:1862–75. 10.1038/nprot.2008.186. 19008873 10.1038/nprot.2008.186 PMC2596932

[31] Mujica E, Hoed M. Investigating the role of lipid genes in liver disease using models of steatotic liver disease in zebrafish (*Danio rerio*). Liver Int. 2023;43(11):2348–50. 10.1111/liv.15752. 37846802 10.1111/liv.15752

[32] Li XX, Ge GD, Song GL, Li Q, Cui ZB. Effects of nutritionally induced obesity on metabolic pathways of zebrafish. Int J Mol Sci. 2023;24(3):1850. 10.3390/ijms24031850. 36768175 10.3390/ijms24031850 PMC9914946

[33] Wang X, Ren YQ, Li J, Ji Z, Chen FS, Wang XD. Identification of the 14-3-3 β/α-A protein as a novel maternal peptidoglycan-binding protein that protects embryos of zebrafish against bacterial infections. Dev Comp Immunol. 2021;114:103867. 10.1016/j.dci.2020.103867. 32931839 10.1016/j.dci.2020.103867

[34] Chen BX, Li C, Wang YD, Lu YA, Wang F, Liu XQ. 14-3-3β/α-A interacts with glycoprotein of spring viremia of carp virus and positively affects viral entry. Fish Shellfish Immunol. 2018;81:438–44. 10.1016/j.fsi.2018.04.031. 29680490 10.1016/j.fsi.2018.04.031

[35] Ding QW, Hao Q, Zhang QS, Yang YL, Olsen RE, RingØ E, et al. DHA suppresses hepatic lipid accumulation via Cyclin D1 in zebrafish. Front Nutr. 2022;8:797510. 10.3389/fnut.2021.797510. 35145984 10.3389/fnut.2021.797510 PMC8823328

[36] Licitra R, Fronte B, Verri T, Marchese M, Sangiacomo C, Santorelli FM. Zebrafish feed intake: a systematic review for standardizing feeding management in laboratory conditions. Biology. 2024;13(4):209. 10.3390/biology13040209. 38666821 10.3390/biology13040209 PMC11047914

[37] Semova I, Carten JD, Stombaugh J, Mackey LC, Knight R, Farber SA, et al. Microbiota regulate intestinal absorption and metabolism of fatty acids in the zebrafish. Cell Host Microbe. 2012;12(3):277–88. 10.1016/j.chom.2012.08.003. 22980325 10.1016/j.chom.2012.08.003 PMC3517662

[38] Zhang Z, Ran C, Ding Qw, Liu HL, Xie MX, Yang YL, et al. Ability of prebiotic polysaccharides to activate a HIF1α-antimicrobial peptide axis determines liver injury risk in zebrafish. Commun Biol. 2019;2:274. 10.1038/s42003-019-0526-z. 31372513 10.1038/s42003-019-0526-z PMC6658494

[39] Valensin D, Cau Y, Calandro P, Vignaroli G, Iacono LD, Chiariello M, et al. Molecular insights to the bioactive form of BV02, a reference inhibitor of 14-3-3σ protein-protein interactions. Bioorg Med Chem Lett. 2016;26(3):894–8. 10.1016/j.bmcl.2015.12.066. 26774582 10.1016/j.bmcl.2015.12.066

[40] Mancini M, Corradi V, Petta S, Barbieri E, Manetti F, Botta M, et al. A new nonpeptidic inhibitor of 14-3-3 induces apoptotic cell death in chronic myeloid leukemia sensitive or resistant to imatinib. J Pharmacol Exp Ther. 2011;336(3):596–604. 10.1124/jpet.110.172536. 21041536 10.1124/jpet.110.172536

[41] Iralde-Lorente L, Cau Y, Clementi L, Franci L, Tassone G, Valensin D, et al. Chemically stable inhibitors of 14-3-3 protein-protein interactions derived from BV02. J Enzyme Inhib Med Chem. 2019;34(1):657–64. 10.1080/14756366.2019.1574779. 30727786 10.1080/14756366.2019.1574779 PMC8853708

[42] Pedroso GL, Hammes TO, Escobar TDC, Fracasso LB, Forgiarini LF, da Silveira TR. Blood collection for biochemical analysis in adult zebrafish. J Vis Exp. 2012;63:e3865. 10.3791/3865. 10.3791/3865 PMC3466942 22664657

[43] Ghosh C, Zhou Y, Collodi P. Derivation and characterization of a zebrafish liver cell line. Cell Biol Toxicol. 1994;10:167–76. 10.1007/BF00757560. 7994634 10.1007/BF00757560

[44] DuBridge RB, Tang P, Hsia HC, Leong PM, Miller JH, Calos MP. Analysis of mutation in human cells by using an Epstein-Barr virus shuttle system. Mol Cell Biol. 1987;7:379–87. 10.1128/mcb.7.1.379. 3031469 10.1128/mcb.7.1.379-387.1987 PMC365079

[45] Folch J, Lees M, Sloane-Stanley GH. A simple method for the isolation and purification of total lipids from animal tissues. J Biol Chem. 1957;226:497–509. 13428781

[46] Wang Y, Wang C, Zhou W, Yang FY, Chen XY, Zhang Q. Effects of wilting and *Lactobacillus plantarum* addition on the fermentation quality and microbial community of *Moringa oleifera* leaf silage. Front Microbiol. 2018;9:1817. 10.3389/fmicb.2018.01817. 30127780 10.3389/fmicb.2018.01817 PMC6087751

[47] Ding Q, Zhang Z, Li Y, Liu H, Hao Q, Yang Y, et al. Propionate induces intestinal oxidative stress via Sod2 propionylation in zebrafish. iScience. 2021;24(6):102515. 10.1016/j.isci.2021.102515. 34142031 10.1016/j.isci.2021.102515 PMC8188496

[48] Wei YW, Zhang SY, Guan GK, Wan ZR, Wang RM, Li PW, et al. A specific and rapid method for detecting *Bacillus* and *Acinetobacter* species in Daqu. Front Bioeng Biotechnol. 2023;11:1261563. 10.3389/fbioe.2023.1261563. 37818237 10.3389/fbioe.2023.1261563 PMC10561003

[49] Ding QW, Hao Q, Jin Y, Zhang QS, Xie YD, Yang YL, et al. The effects of sodium propionate on intestinal barrier function of genetically improved farmed tilapia in a high-lipid formulation. Aquaculture. 2024;579:740187. 10.1016/j.aquaculture.2023.740187.

[50] Guo W, Lei L, Shi XJ, Li RW, Wang QW, Han J, et al. Nonalcoholic fatty liver disease development in zebrafish upon exposure to bis(2-ethylhexyl)-2,3,4,5-tetrabromophthalate, a novel brominated flame retardant. Environ Sci Technol. 2021;55(10):6926–35. 10.1021/acs.est.1c01476. 33938212 10.1021/acs.est.1c01476

[51] Dong YZ, Yu MH, Wu YL, Xia T, Wang L, Song K, et al. Hydroxytyrosol promotes the mitochondrial function through activating mitophagy. Antioxidants. 2022;11(5):893. 10.3390/antiox11050893. 35624756 10.3390/antiox11050893 PMC9138034

[52] Ipsen DH, Lykkesfeldt J, Tveden-Nyborg P. Molecular mechanisms of hepatic lipid accumulation in non-alcoholic fatty liver disease. Cell Mol Life Sci. 2018;75:3313–27. 10.1007/s00018-018-2860-6. 29936596 10.1007/s00018-018-2860-6 PMC6105174

[53] Morán-Salvador E, López-Parra M, García-Alonso V, Titos E, Martínez-Clemente M, González-Périz A, et al. Role for PPARγ in obesity-induced hepatic steatosis as determined by hepatocyte- and macrophage-specific conditional knockouts. FASEB J. 2011;25(8):2538–50. 10.1096/fj.10-173716. 21507897 10.1096/fj.10-173716

[54] Nguyen P, Valanejad L, Cast A, Wright M, Garcia JM, El-Serag HB, et al. Elimination of age-associated hepatic steatosis and correction of aging phenotype by inhibition of cdk4-C/EBPα-p300 axis. Cell Rep. 2018;24(6):1597–609. 10.1016/j.celrep.2018.07.014. 30089269 10.1016/j.celrep.2018.07.014 PMC8209958

[55] Grabner GF, Xie H, Schweiger M, Zechner R. Lipolysis: cellular mechanisms for lipid mobilization from fat stores. Nat Metab. 2021;3:1445–65. 10.1038/s42255-021-00493-6. 34799702 10.1038/s42255-021-00493-6

[56] Turpin SM, Hoy AJ, Brown RD, Rudaz CG, Honeyman J, Matzaris M, et al. Adipose triacylglycerol lipase is a major regulator of hepatic lipid metabolism but not insulin sensitivity in mice. Diabetologia. 2011;54:146–56. 10.1007/s00125-010-1895-5. 20842343 10.1007/s00125-010-1895-5

[57] Grasselli E, Voci A, Demori I, Vecchione G, Compalati AD, Gallo G, et al. Triglyceride mobilization from lipid droplets sustains the anti-steatotic action of iodothyronines in cultured rat hepatocytes. Front Physiol. 2016;6:418. 10.3389/fphys.2015.00418. 26793120 10.3389/fphys.2015.00418 PMC4709507

[58] Wu Y, Du HH, Zhu L, Zhao N, Zhang SN, Cao ZJ, et al. Bactericidal permeability-increasing protein/LPS-binding protein (BPI/LBP) enhances resistance of golden pompano *Trachinotus ovatus* against bacterial infection. Fish Shellfish Immunol. 2022;131:872–80. 10.1016/j.fsi.2022.10.065. 36347416 10.1016/j.fsi.2022.10.065

[59] Milosevic I, Vujovic A, Barac A, Djelic M, Korac M, Spurnic AR, et al. Gut-liver axis, gut microbiota, and its modulation in the management of liver diseases: a review of the literature. Int J Mol Sci. 2019;20(2):395. 10.3390/ijms20020395. 30658519 10.3390/ijms20020395 PMC6358912

[60] Aron-Wisnewsky J, Vigliotti C, Witjes J, Le P, Holleboom AG, Verheij J, et al. Gut microbiota and human NAFLD: disentangling microbial signatures from metabolic disorders. Nat Rev Gastro Hepat. 2020;17:279–97. 10.1038/s41575-020-0269-9. 10.1038/s41575-020-0269-9 32152478

[61] Yang ZD, Su HH, Lv YJ, Tao HQ, Jiang YH, Ni ZY, et al. Inulin intervention attenuates hepatic steatosis in rats via modulating gut microbiota and maintaining intestinal barrier function. Food Res Int. 2023;163:112309. 10.1016/j.foodres.2022.112309. 36596207 10.1016/j.foodres.2022.112309

[62] Yang ZD, Su HH, Chen XQ, Ni ZY, Tao HQ, Jiang YH, et al. Isomalt attenuates hepatic steatosis in rats via modulating gut microbiota and its metabolic function. J Funct Foods. 2024;112:105963. 10.1016/j.jff.2023.105963. 10.1016/j.foodres.2022.112309 36596207

[63] Falcinelli S, Rodiles A, Hatef A, Picchietti S, Cossignani L, Merrifield DL, et al. Dietary lipid content reorganizes gut microbiota and probiotic *L. rhamnosus* attenuates obesity and enhances catabolic hormonal milieu in zebrafish. Sci Rep. 2017;7:5512. 10.1038/s41598-017-05147-w. 28717234 10.1038/s41598-017-05147-w PMC5514052

[64] Sui GY, Jia LQ, Quan DM, Zhao N, Yang GL. Activation of the gut microbiota-kynurenine-liver axis contributes to the development of nonalcoholic hepatic steatosis in nondiabetic adults. Aging. 2021;13(17):21309–24. 10.18632/aging.203460. 34473644 10.18632/aging.203460 PMC8457600

[65] Wang QY, Huang J, Liu S, Wang CY, Jin YX, Lai H, et al. Aberrant hepatic lipid metabolism associated with gut microbiota dysbiosis triggers hepatotoxicity of novel PFOS alternatives in adult zebrafish. Environ Int. 2022;166:107351. 10.1016/j.envint.2022.107351. 35738203 10.1016/j.envint.2022.107351

[66] Liu Y, Zhu DX, Liu JW, Sun XX, Gao F, Duan HP, et al. *Pediococcus pentosaceus* PR-1 modulates high-fat-died-induced alterations in gut microbiota, inflammation, and lipid metabolism in zebrafish. Front Nutr. 2023;10:1087703. 10.3389/fnut.2023.1087703. 36819708 10.3389/fnut.2023.1087703 PMC9929557

[67] Ren ZH, Okyere SK, Xie L, Wen J, Wang JY, Chen ZL, et al. Oral administration of *Bacillus toyonensis* strain SAU-20 improves insulin resistance and ameliorates hepatic steatosis in type 2 diabetic mice. Front Immunol. 2022;13:837237. 10.3389/fimmu.2022.837237. 35242140 10.3389/fimmu.2022.837237 PMC8887768

[68] Zhang FL, Luan YY, Hao Q, Zhang QS, Yang YL, Ran C, et al. Nuclease treatment enhances the probiotic effect of *Bacillus velezensis* T23 on hepatic steatosis and inflammation induced by high-fat diet in zebrafish. Aquaculture. 2023;562:738801. 10.1016/j.aquaculture.2022.738801.

[69] Zhang J, Zhou J, He ZY, Li HS. *Bacteroides* and NAFLD: pathophysiology and therapy. Front Microbiol. 2024;15:1288856. 10.3389/fmicb.2024.1288856. 38572244 10.3389/fmicb.2024.1288856 PMC10988783

[70] Lu S, Na K, Li YR, Zhang L, Fang Y, Guo XH. *Bacillus*-derived probiotics: metabolites and mechanisms involved in bacteria–host interactions. Crit Rev Food Sci. 2024;64(6):1701–14. 10.1080/10408398.2022.2118659. 10.1080/10408398.2022.2118659 36066454

[71] Catlett JL, Catazaro J, Cashman M, Carr S, Powers R, Cohen MB, et al. Metabolic feedback inhibition influences metabolite secretion by the human gut symbiont *Bacteroides thetaiotaomicron*. Msystems. 2020;5(5):e00252-20. 10.1128/msystems.00252-20. 32873608 10.1128/mSystems.00252-20 PMC7470985

[72] He JM, Hou TY, Wang QW, Wang QY, Jiang Y, Chen LY, et al. L-Arginine metabolism ameliorates age-related cognitive impairment by Amuc_1100-mediated gut homeostasis maintaining. Aging Cell. 2024;23(4):e14081. 10.1111/acel.14081. 38236004 10.1111/acel.14081 PMC11019123

[73] Sellmann C, Degen C, Jin CJ, Nier A, Engstler AJ, Alkhatib DH, et al. Oral arginine supplementation protects female mice from the onset of non-alcoholic steatohepatitis. Amino Acids. 2017;49:1215–25. 10.1007/s00726-017-2423-4. 28434046 10.1007/s00726-017-2423-4 PMC5487836

[74] Chen QC, Fang W, Shen YA, Xu D, Chen Q, Cui K, et al. Suppression of cideb under endoplasmic reticulum stress exacerbated hepatic inflammation by inducing hepatic steatosis and oxidative stress. Free Radic Biol Med. 2022;185:67–75. 10.1016/j.freeradbiomed.2022.04.009. 35489563 10.1016/j.freeradbiomed.2022.04.009

[75] Wang HY, Su SG, An X, Xu Y, Sun JC, Zhen MM, et al. A charge reversal nano-assembly prevents hepatic steatosis by resolving inflammation and improving lipid metabolism. Bioact Mater. 2025;45:496–508. 10.1016/j.bioactmat.2024.11.023. 39717365 10.1016/j.bioactmat.2024.11.023 PMC11664292

[76] Burns AR, Stephens WZ, Stagaman K, Wong S, Rawls JF, Guillemin K, et al. Contribution of neutral processes to the assembly of gut microbial communities in the zebrafish over host development. ISME J. 2016;10(3):655–64. 26296066 10.1038/ismej.2015.142 PMC4817674

[77] Stagaman K, Burns AR, Guillemin K, Bohannan BJ. The role of adaptive immunity as an ecological filter on the gut microbiota in zebrafish. ISME J. 2017;11(7):1630–9. 10.1038/ismej.2017.28. 10.1038/ismej.2017.28 PMC5520148 28304369

[78] Roeselers G, Mittge EK, Stephens WZ, Parichy DM, Cavanaugh CM, Guillemin K, et al. Evidence for a core gut microbiota in the zebrafish. ISME J. 2011;5(10):1595–608. 21472014 10.1038/ismej.2011.38 PMC3176511

[79] Wong S, Stephens WZ, Burns AR, Stagaman K, David LA, Bohannan BJ, et al. Ontogenetic differences in dietary fat influence microbiota assembly in the zebrafish gut. MBio. 2015;6(5):e00687-15. 26419876 10.1128/mBio.00687-15 PMC4611033

[80] Zinöcker MK, Lindseth IA. The Western diet–microbiome-host interaction and its role in metabolic disease. Nutrients. 2018;10(3):365. 29562591 10.3390/nu10030365 PMC5872783

[81] Zhang J, Kong XH, Zhou CJ, Li L, Nie GX, Li XJ. Toll-like receptor recognition of bacteria in signal pathways fish: ligand specificity and signal pathways. Fish Shellfish Immunol. 2014;41(2):380–8. 10.1016/j.fsi.2014.09.022. 25241605 10.1016/j.fsi.2014.09.022

[82] Abdrabou A, Brandwein D, Wang ZX. Differential subcellular distribution and translocation of seven 14-3-3 isoforms in response to EGF and during the cell cycle. Int J Mol Sci. 2020;21(1):318. 10.3390/ijms21010318. 31906564 10.3390/ijms21010318 PMC6981507

[83] van Heusden GPH. 14-3-3 proteins: regulators of numerous eukaryotic proteins. IUBMB Life (International Union of Biochemistry and Molecular Biology: Life). 2005;57(9):623–9. 10.1080/15216540500252666. 16203681 10.1080/15216540500252666

[84] Muslin AJ, Xing HM. 14-3-3 proteins: regulation of subcellular localization by molecular interference. Cell Signal. 2000;12(11–12):703–9. 10.1016/S0898-6568(00)00131-5. 11152955 10.1016/s0898-6568(00)00131-5

[85] Root A, Beizaei A, Ebhardt HA. Structure-based assessment and network analysis of targeting 14-3-3 proteins in prostate cancer. Mol Cancer. 2018;17(1):156. 30382885 10.1186/s12943-018-0905-y PMC6208026

[86] Cosse M, Seidel T. Plant proton pumps and cytosolic pH-homeostasis. Front Plant Sci. 2021;12:672873. 10.3389/fpls.2021.672873. 34177988 10.3389/fpls.2021.672873 PMC8220075

[87] Subbarayudu S, Snega Priya P, Rajagopal R, Alfarhan A, Guru A, Arockiaraj J. Impact of acidic and alkaline conditions on *Staphylococcus aureus* and *Acinetobacter baumannii* interactions and their biofilms. Arch Microbiol. 2024;206(11):426. 10.1007/s00203-024-04142-w 39375235

[88] Luu S, Setlow P. Analysis of the loss in heat and acid resistance during germination of spores of *Bacillus* species. J Bacteriol. 2014;196(9):1733–40. 24563034 10.1128/JB.01555-14 PMC3993331

[89] Thavornjikarn K. Survey of some enteropathogenic bacteria in Thai fermented fish (*Som-fug*). Kasetsart University, Master's thesis. 1985.

[90] Namdari H, Cabelli VJ. The suicide phenomenon in motile aeromonads. Appl Environ Microbiol. 1989;55(3):543–7. 10.1128/aem.55.3.543-547.1989. 2930167 10.1093/benz/9780199773787.article.b00013623 PMC184157

[91] Wang JC, Xu WJ, Wang RJ, Cheng RR, Tang ZQ, Zhang M. The outer membrane protein Amuc_1100 of *Akkermansia muciniphila* promotes intestinal 5-HT biosynthesis and extracellular availability through TLR2 signalling. Food Funct. 2021;12:3597–610. 10.1039/D1FO00115A. 33900345 10.1039/d1fo00115a

[92] Wu XH, Yu DH, Ma YK, Fang XX, Sun PD. Function and therapeutic potential of Amuc_1100, an outer membrane protein of *Akkermansia muciniphila*: A review. Int J Biol Macromol. 2025;308(4):142442. 10.1016/j.ijbiomac.2025.142442. 40157674 10.1016/j.ijbiomac.2025.142442

[93] Kuebutornye FKA, Abarike ED, Lu YS, Hlordzi V, Sakyi ME, Afriyie G, et al. Mechanisms and the role of probiotic in mitigating fish pathogens in aquaculture. Fish Physiol Biochem. 2020;46:819–841.10.1007/s10695-019-00754-y. 10.1007/s10695-019-00754-y 31953625

[94] Lalloo R, Moonsamy G, Ramchuran S, Görgens J, Gardiner N. Competitive exclusion as a mode of action of a novel *Bacillus cereus* aquaculture biological agent. Lett Appl Microbiol. 2010;50(6):563–70. 10.1111/j.1472-765X.2010.02829.x. 20337929 10.1111/j.1472-765X.2010.02829.x

[95] Eishingdrelo H, Qin X, Yuan L, Kongsamut S, Yu L. Ligands can differentially and temporally modulate GPCR interaction with 14-3-3 isoforms. Curr Res Pharmacol Drug Discov. 2022;3:100123. 10.1016/j.crphar.2022.100123. 35992381 10.1016/j.crphar.2022.100123 PMC9389249

[96] Sung Y, Choi Y, Kim ES, Ryu JH, Kwon IC. Receptor-ligand interactions for optimized endocytosis in targeted therapies. J Control Release. 2025;380:524–38. 10.1016/j.jconrel.2025.01.060. 39875075 10.1016/j.jconrel.2025.01.060

[97] Swaan PW. Recent advances in intestinal macromolecular drug delivery via receptor-mediated transport pathways. Pharm Res-Dordr. 1998;15:826–34. 10.1023/A:1011908128045. 10.1023/a:1011908128045 9647346

