# Supplementary material for: Amuc_1100 alleviates HFD-induced hepatic lipid accumulation via gut microbiota in zebrafish: insights from the role of intestinal 14-3-3β/α-A
Source: J Anim Sci Biotechnol. 2026 Jun 10;17:115. doi: 10.1186/s40104-026-01418-7 (PMC13251306; doi:10.1186/s40104-026-01418-7)
Supplement: Supplementary file 1 — Additional file 1: Fig. S1 Effects of Amuc_1100 on the growth performance and hepatic TAG of 1-month-old zebrafish, and intracellular TAG accumulation in ZFL cells. Fig. S2 Effects of Amuc_1100 on hepatic steatosis in GF zebrafish. Fig. S3 PERMANOVA at the OTU level among the LFD, HFD, and AM0.01 groups after a 4-week feeding period. Fig. S4 Effects of Amuc_1100 on fish gut-derived strains ex vivo. Fig. S5 PERMANOVA at the OTU level among the LFD-BV02, HFD-BV02, and AM0.01-BV02 groups after a 4-week feeding period. Fig. S6 Effects of LFD-BV02, HFD-BV02 or AM0.01-BV02 gut microbiota on HFD-induced hepatic steatosis in GF zebrafish. Fig. S7 Representative images showing the intracellular location of exogenous Amuc_1100-GFP in ZF4 cells. Table S1 Ingredients for 1-month-old zebrafish diet (g/kg dry diet). Table S2 Ingredients for zebrafish larvae diet at 5 dpf (g/kg dry diet). Table S3 Sequences of primers. Table S4 Fragments that identified by mass spectrometry after digestion in vitro. Table S5 Predicted amino acid residues involving the interaction between the Amuc_1100 and 14-3-3β/α-A. [file 40104_2026_1418_MOESM1_ESM.docx]

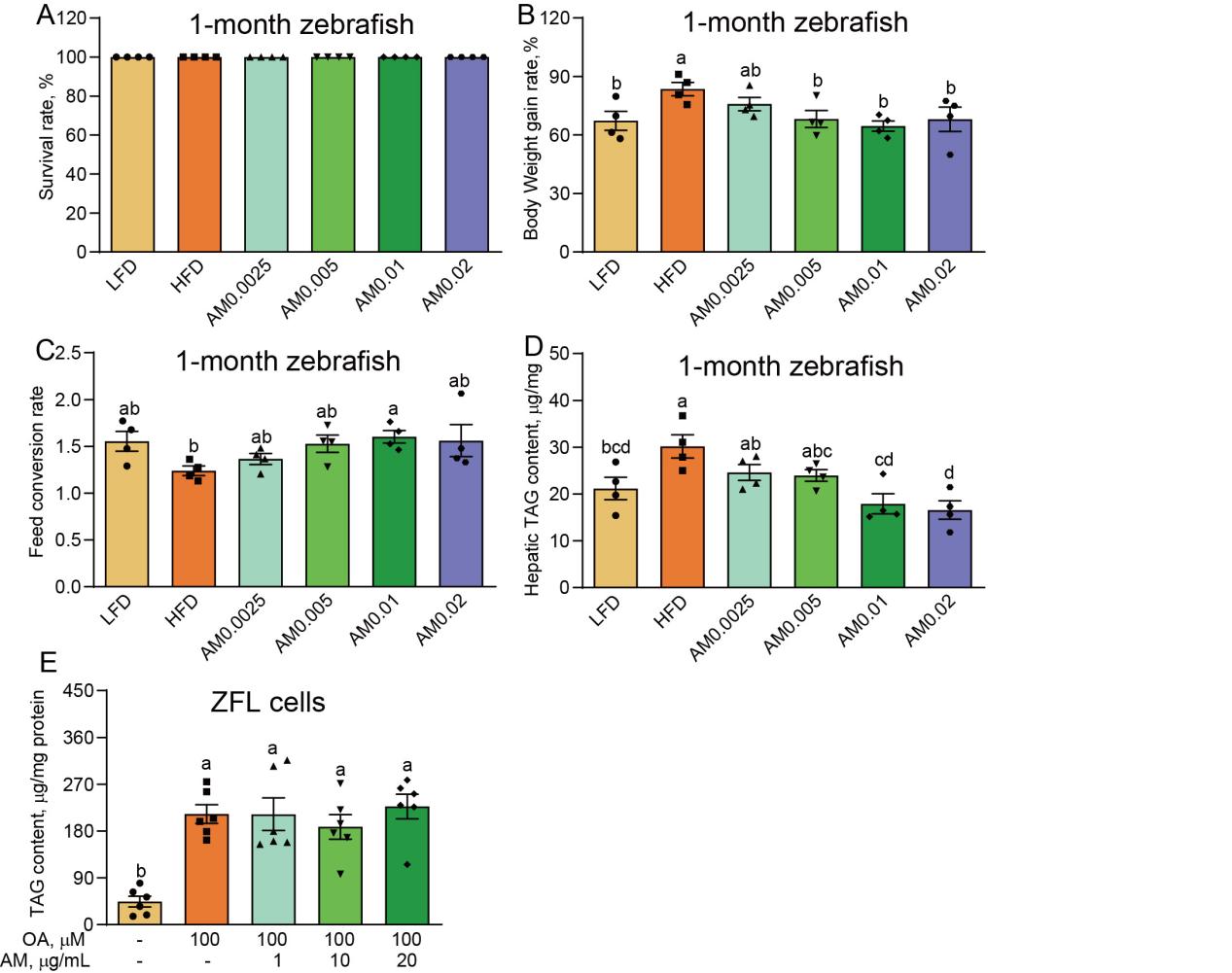


**Fig. S1** Effects of Amuc_1100 on the growth performance and hepatic TAG of 1-month-old zebrafish, and intracellular TAG accumulation in ZFL cells. **A**−**D** The effects of Amuc_1100 on survival rate (**A**), body weight gain (**B**), feed conversion ratio (**C**), and hepatic TAG (**D**) of 1-month-old zebrafish fed the LFD, HFD and Amuc_1100-supplemented HFDs for 4 weeks (*n* = 4, 1 tank per replicate). **E** TAG contents in ZFL cells treated with OA or OA plus Amuc_1100 for 24 h (*n* = 6). Data are expressed as mean ± SEM. Means without a common letter are significantly different (*P* < 0.05). Duncan’s test


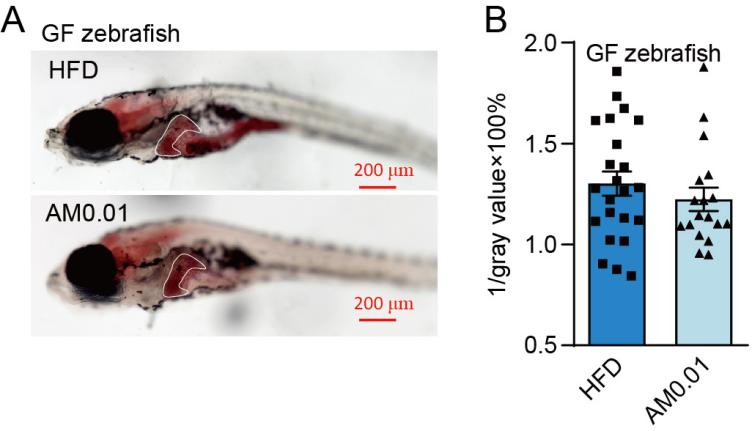


**Fig. S2** Effects of Amuc_1100 on hepatic steatosis in GF zebrafish. **A** and **B** Zebrafish at 5 dpf fed on the LFD, HFD and AM0.01 for 7 d. **A** Representative images of whole-mount ORO staining. The scale bar = 200 μm. **B** Quantitative assessment of whole-mount ORO staining (*n* ≥ 18). Data are expressed as mean ± SEM. Student’s *t*-test


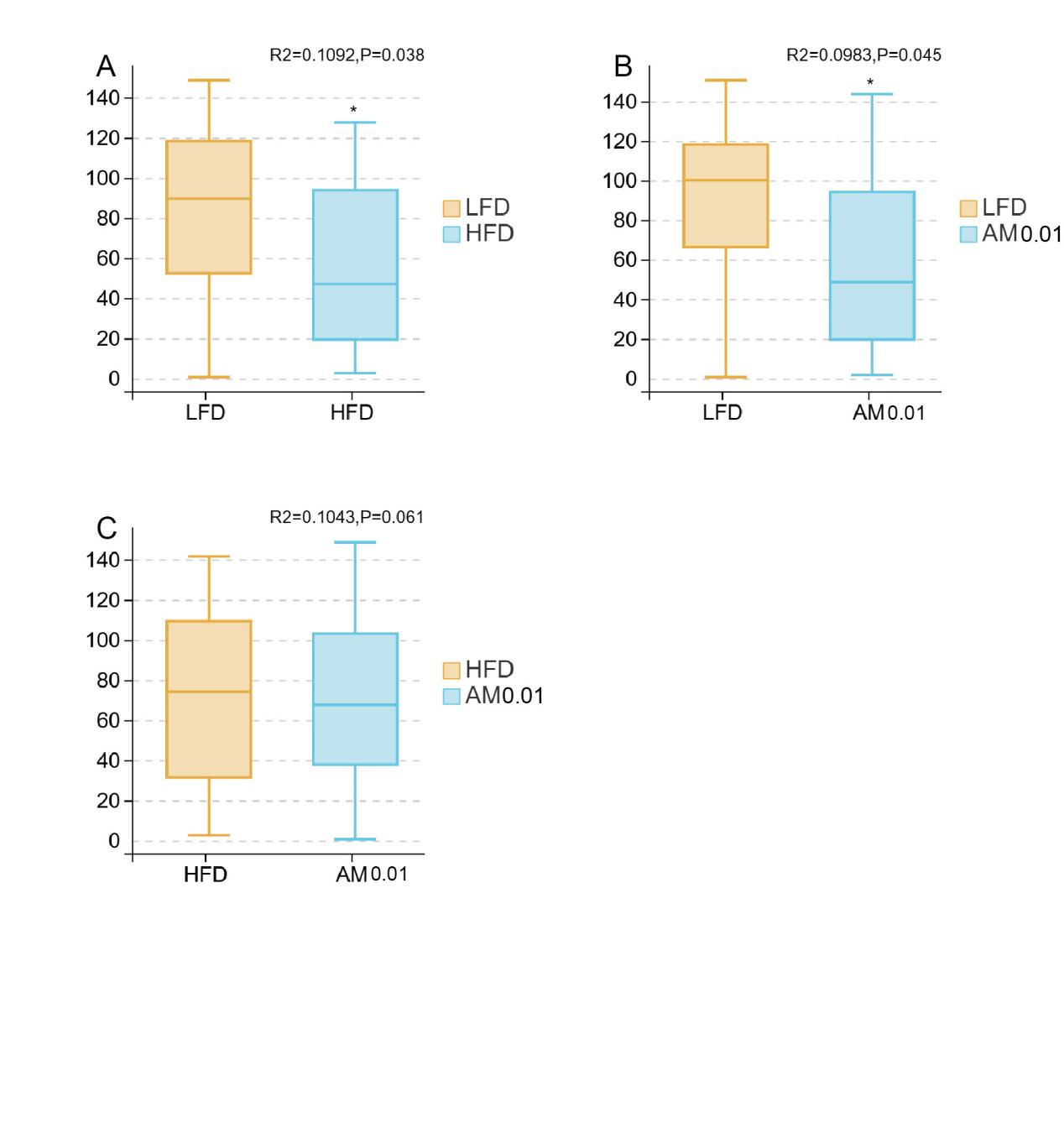


**Fig. S3** PERMANOVA at the OTU level between the LFD, HFD, and AM0.01 groups after a 4-week feeding period (*n* = 9). Data are expressed as mean ± SD. ^*^*P* < 0.05


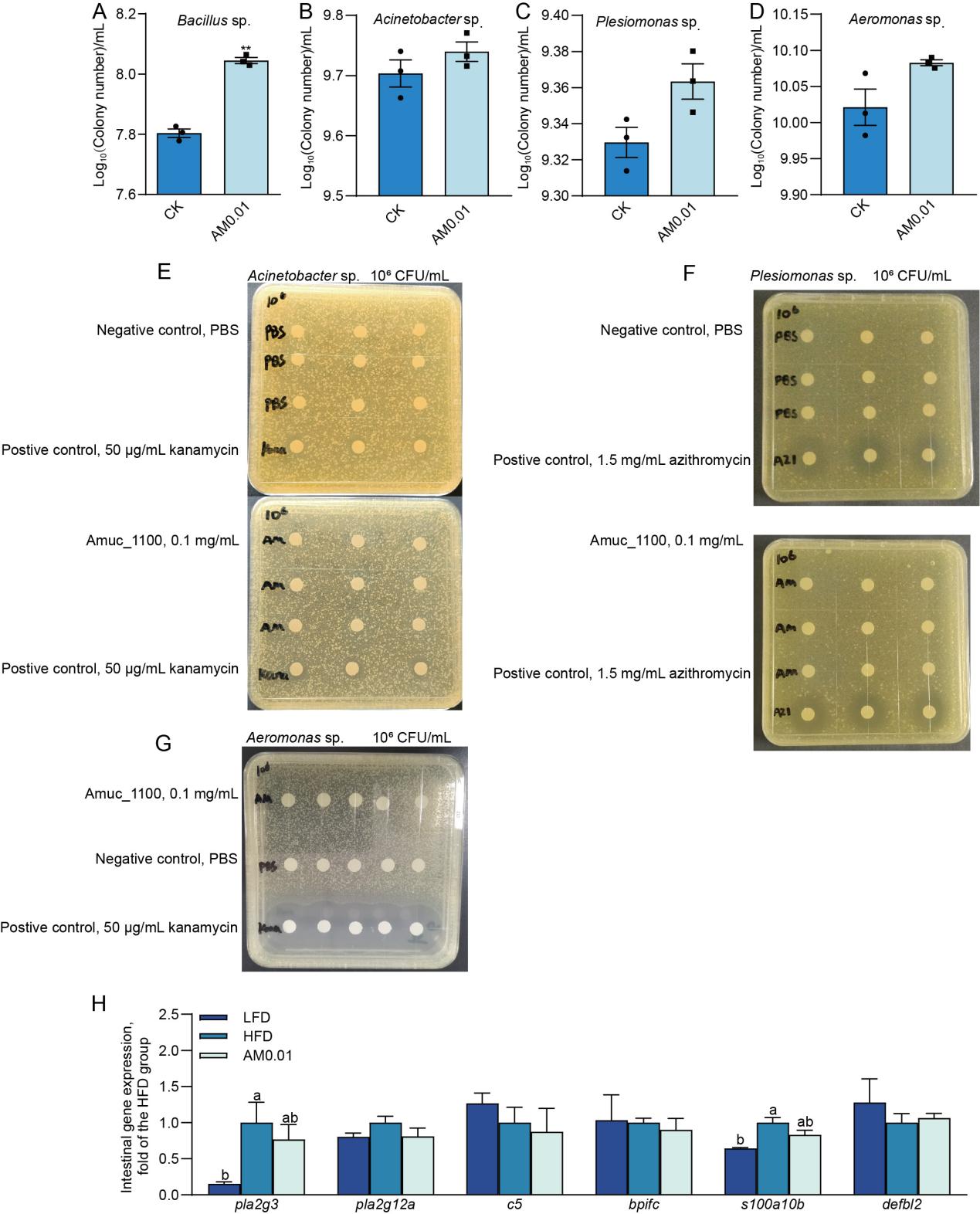


**Fig. S4** Effects of Amuc_1100 on fish gut-derived strains ex vivo. **A**−**D** The concentration of fish gut-derived *Bacillus* (**A**), *Acinetobacter* (**B**), *Plesiomonas* (**C**) and *Aeromonas* (**D**) strains after culturing in Amuc_1100-supplemented medium for 18 h (*n* = 3). **E**−**G** Images showing inhibition of PBS, Amuc_1100 or specific antibiotics to fish gut-derived *Acinetobacter* (**A**), *Plesiomonas* (**B**) and *Aeromonas* (**C**) strains. **H** The relative mRNA expression of genes encoding antibacterial proteins targeting Gram-negative bacteria (*pla2g3*, *pla2g12a*, *c5*, *bpifc*, *s100a10b*) and antimicrobial peptides (*hepcidin* and *defbl2*) on the intestine (*n* = 3). Data are expressed as mean ± SEM. **A**−**D** ^**^*P* < 0.01. Student’s *t*-test. **H** Means without a common letter are significantly different (*P* < 0.05). Duncan’s test


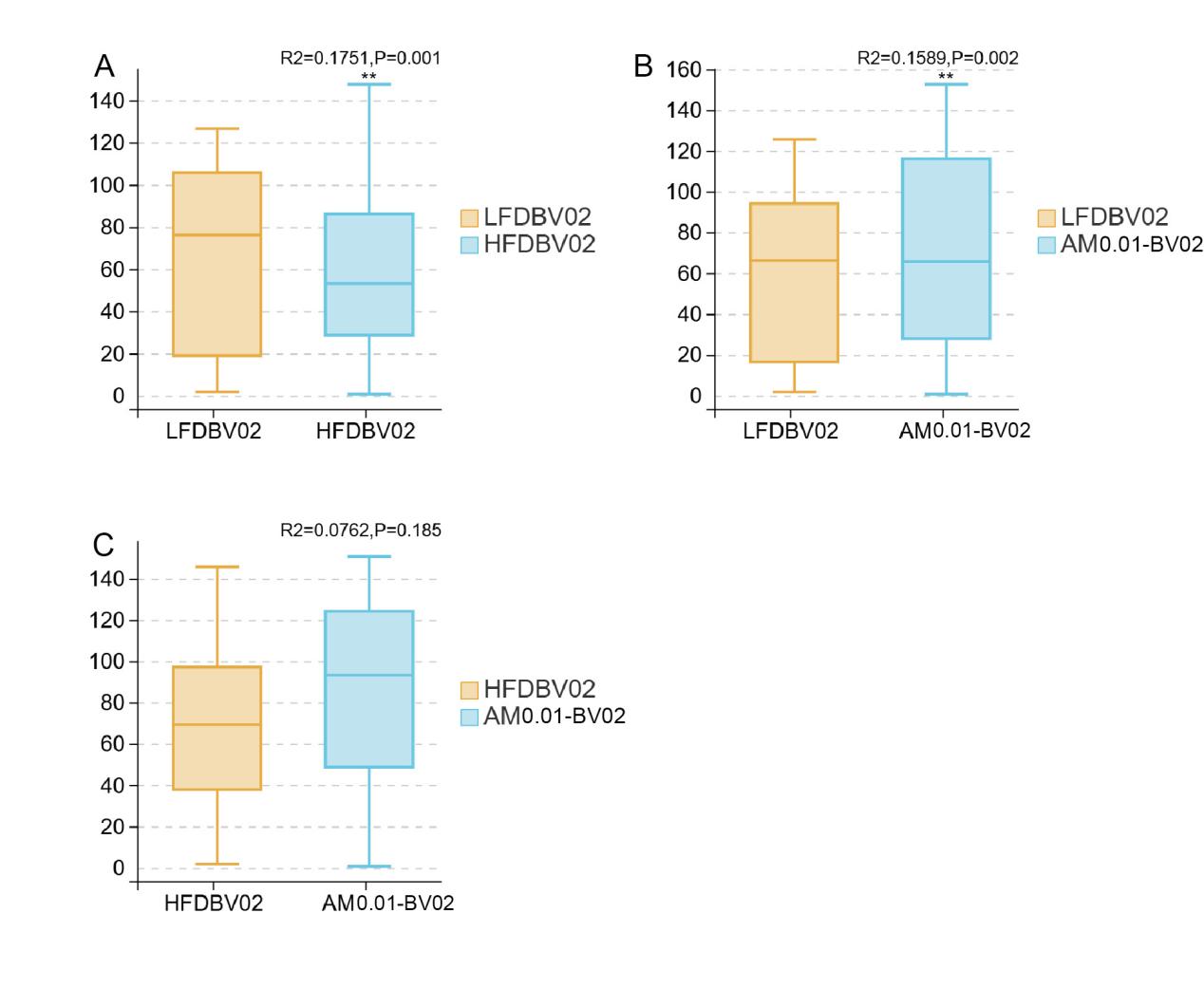


**Fig. S5** PERMANOVA at the OTU level between the LFD-BV02, HFD-BV02, and AM0.01-BV02 groups after a 4-week feeding period (*n* = 9). Data are expressed as mean ± SD. ^**^*P* < 0.01


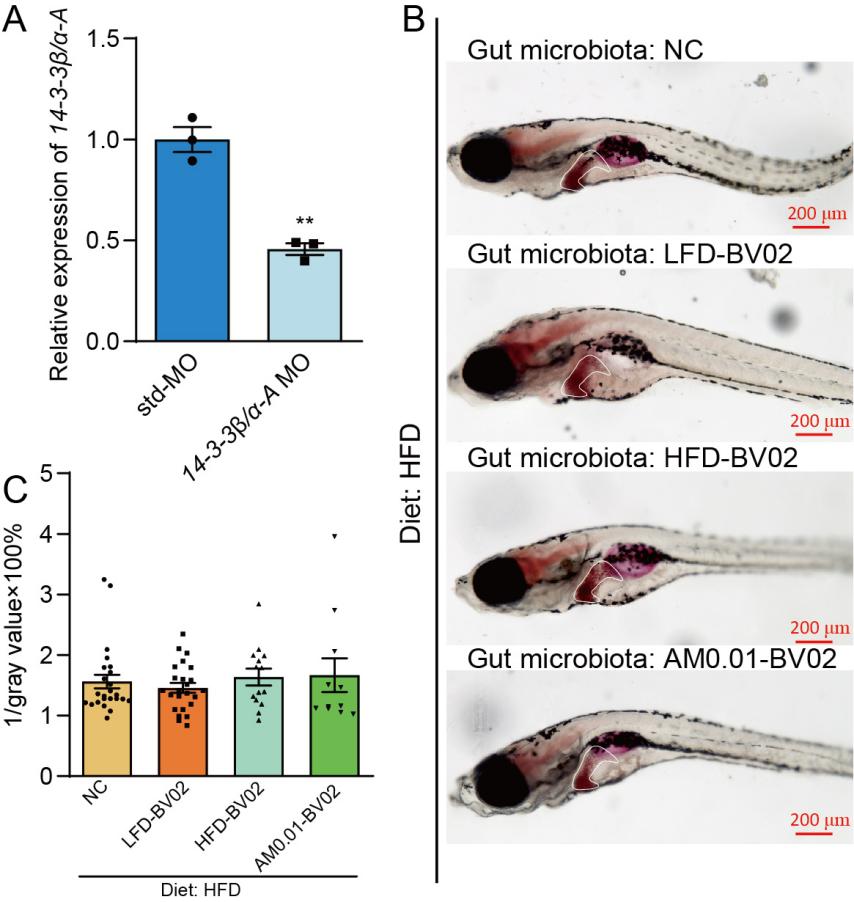


**Fig. S6** Effects of LFD-BV02, HFD-BV02 or HFAM0.01-BV02 gut microbiota on HFD-induced hepatic steatosis in GF zebrafish. **A** The efficiency of *14-3-3β/α-A* vivo-MO in zebrafish larvae (*n* = 3). **B** and **C** GF zebrafish at 5 dpf fed the HFD for 7 d, and were inoculated with gut microbiota from one-month-old zebrafish fed the LFD-BV02, HFD-BV02 or HFAM0.01-BV02. **B** Representative images of whole-mount ORO staining. The scale bar = 200 μm. **C** Quantitative assessment of whole-mount ORO staining (*n* ≥ 11). Data are expressed as mean ± SEM. **A** ^**^*P* < 0.01. Student’s *t*-test. **C** Duncan’s test


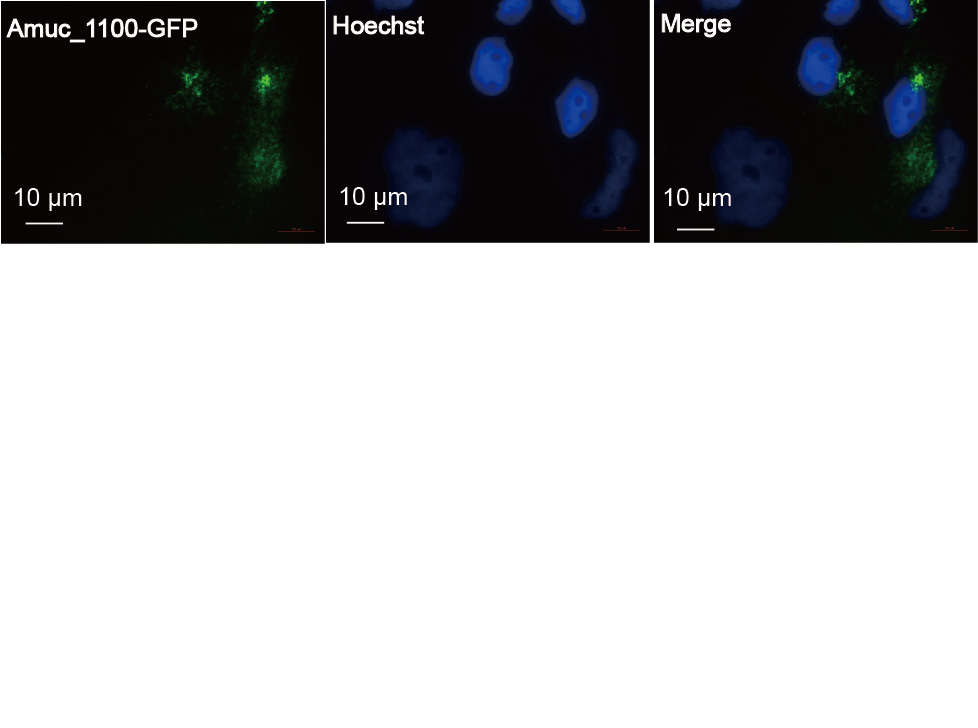


**Fig. S7** Representative images showing the intracellular location of exogenous Amuc_1100-GFP in ZF4 cells. The scale bar = 10 μm

**Table S1** Ingredients for 1-month-old zebrafish diet (g/kg dry diet)

|  | **1-month-old zebrafish** | | | | | |
| --- | --- | --- | --- | --- | --- | --- |
| **Ingredients**  **(g/kg dry diet)** | **LFD** | **HFD** | **AM0.0025** | **AM0.005** | **AM0.01** | **AM0.02** |
| Casein | 400 | 400 | 399.975 | 399.95 | 399.9 | 399.8 |
| Gelatin | 100 | 100 | 100 | 100 | 100 | 100 |
| Wheat flour | 280 | 160 | 160 | 160 | 160 | 160 |
| Amuc_1100 | 0 | 0 | 0.025 | 0.05 | 0.1 | 0.2 |
| Lard | 30 | 80 | 80 | 80 | 80 | 80 |
| Soybean oil | 30 | 80 | 80 | 80 | 80 | 80 |
| Lysine | 3.3 | 3.3 | 3.3 | 3.3 | 3.3 | 3.3 |
| VC phosphate | 1 | 1 | 1 | 1 | 1 | 1 |
| Vitamin premix^1^ | 4 | 4 | 4 | 4 | 4 | 4 |
| Mineral premix^2^ | 4 | 4 | 4 | 4 | 4 | 4 |
| Monocalcium phosphate | 20 | 20 | 20 | 20 | 20 | 20 |
| Choline chloride | 2 | 2 | 2 | 2 | 2 | 2 |
| Sodium alginate | 20 | 20 | 20 | 20 | 20 | 20 |
| Microcrystalline Cellulose | 40 | 40 | 40 | 40 | 40 | 40 |
| Zeolite | 65.7 | 85.7 | 85.7 | 85.7 | 85.7 | 85.7 |
| Total | 1000 | 1000 | 1000 | 1000 | 1000 | 1000 |
| Proximate composition  (g/kg dry diet) |  |  |  |  |  |  |
| Crude protein | 449.4 | 430.9 | 431.1 | 430.7 | 430.7 | 431.2 |
| Crude lipid | 43.9 | 157.0 | 157.1 | 157.3 | 159.9 | 157.1 |
| Ash | 69.2 | 61.0 | 60.9 | 60.9 | 61.0 | 61.3 |

^1^Vitamin premix (g/kg): thiamine, 0.438; riboflavin, 0.632; pyridoxine·HCl, 0.908; *d*-pantothenic acid, 1.724; nicotinic acid, 4.583; biotin, 0.211; folic acid, 0.549; vitamin B-12, 0.001; inositol, 21.053; menadione sodium bisulfite, 0.889; retinyl acetate, 0.677; cholecalciferol, 0.116; *dl*-α-tocopherol-acetate, 12.632

^2^Mineral premix (g/kg): CoCl_2_·6H_2_O, 0.074; CuSO_4_·5H_2_O, 2.5; FeSO_4_·7H_2_O, 73.2; NaCl, 40.0; MgSO_4_·7H_2_O, 284.0; MnSO_4_·H_2_O, 6.50; KI, 0.68; Na_2_SeO_3_, 0.10; ZnSO_4_·7H_2_O, 131.93; Cellulose, 501.09

**Table S2** Ingredients for zebrafish larvae diet at 5 dpf (g/kg dry diet)

| **Ingredients**  **(g/kg dry diet)** | **5 dpf zebrafish larva** | | |
| --- | --- | --- | --- |
|  | **LFD** | **HFD** | **AM0.01** |
| Casein | 460 | 460 | 459.9 |
| Geltin | 110 | 110 | 110 |
| Wheat flour | 220 | 100 | 100 |
| Lard | 0 | 80 | 80 |
| Soybean oil | 35 | 80 | 80 |
| Cod liver oil | 35 | 40 | 40 |
| Amuc_1100 | 0 | 0 | 0.1 |
| Soybean lecithin | 20 | 20 | 20 |
| Lysine | 3.7 | 3.7 | 3.7 |
| VC phosphate | 1 | 1 | 1 |
| Vitamin premix^1^ | 4 | 4 | 4 |
| Mineral premix^2^ | 4 | 4 | 4 |
| Monocalcium phosphate | 20 | 20 | 20 |
| Choline chloride | 2 | 2 | 2 |
| Sodium alginate | 40 | 40 | 40 |
| Zeolite | 45.3 | 35.3 | 35.3 |
| Total | 1000 | 1000 | 1000 |

^1^Vitamin premix (g/kg): thiamine, 0.438; riboflavin, 0.632; pyridoxine·HCl, 0.908; *d*-pantothenic acid, 1.724; nicotinic acid, 4.583; biotin, 0.211; folic acid, 0.549; vitamin B-12, 0.001; inositol, 21.053; menadione sodium bisulfite, 0.889; retinyl acetate, 0.677; cholecalciferol, 0.116; *dl*-α-tocopherol-acetate, 12.632

^2^Mineral premix (g/kg): CoCl_2_·6H_2_O, 0.074; CuSO_4_·5H_2_O, 2.5; FeSO_4_·7H_2_O, 73.2

**Table S3** Sequences of primers

| **Primers** | **Sense (5’-3’)** | **Antisense (5’-3’)** | **Product size** | **Reference** |
| --- | --- | --- | --- | --- |
| *rps11* | ACAGAAATGCCCCTTCACTG | GCCTCTTCTCAAAACGGTTG | 146 | [38] |
| *pparγ* | CCTGTCCGGGAAGACCAGCG | GTGCTCGTGGAGCGGCATGT | 109 | [38] |
| *srebf1* | CAGAGGGTGGGCATGCTGGC | ATGTGACGGTGGTGCCGCTG | 118 | [38] |
| *c/ebpα* | GCCGCATCTGTCCTACCTT | TGTTTCTTGGATTTCCCTCG | 174 | This study |
| *fas* | GGAGCAGGCTGCCTCTGTGC | TTGCGGCCTGTCCCACTCCT | 128 | [38] |
| *acc1* | GCGTGGCCGAACAATGGCAG | GCAGGTCCAGCTTCCCTGCG | 137 | [38] |
| *dgat2* | CCTTACACGACCTGCCCAC | CAAAAAGCCCCAAAACACAA | 370 | This study |
| *atgl* | GCAAGGAGTGAGGTATGTGGA | GTAGAGGTTGGCGAGTGTGAA | 187 | This study |
| *cpt1aa* | GCCTTTCAGTTCACCGTCG | CGCTTCCTGGATATACCCC | 151 | This study |
| *14-3-3β/α-A* | TGATGACATGGCTGCGTCTATGAAG | TTCTGCTCAATGCTGGAGATCACAC | 147 | This study |
| *Acinetobacter* | ATGTGAAATCCCCGAGCTT | AGTTTGTCACTGGCAGTATCCT | 572 | [48] |
| *Plesiomonas* | CTCCGAATACCGTAGAGTGCTATCC | CTCCCCTAGCCCAATAACACCTAAA | 284 | [38] |
| *Aeromonas* | GAAGGCCAAGTCGGCCGCCAG | ATCTTGGCACGCCCGGGTTTTC | 197 | This study |
| *Bacillus* | GTCTGTAACTGACGCTGAGGC | GCGATTACTAGCGATTCCA | 614 | [48] |

**Table S4** Fragments that identified by mass spectrometry after digestion in vitro

| **Fragments** | **N-terminal** | **C-terminal** |
| --- | --- | --- |
| I31–K119 | IVNSKRSELD | FRDSLISSCK |
| I43–Y137 | ISIAAK | QVY |
| S138–A317 | STQAPSVQAASTLGFELK | AQEPSED |

**Table S5** Predicted amino acid residues involving the interaction between the Amuc_1100 and protein 14-3-3β/α-A

| **Amuc_1100** | | **14-3-3β/α-A** | |
| --- | --- | --- | --- |
| R.**NERMM**.P | N240 | M.**Q**LL**RD**NL**T** | Q219, R222 |
|  | E241 |  | R222 |
|  | R242 |  | Q219, R222, T226 |
|  | M243 |  | Q219 |
|  | M244 |  | D223 |
| A.**Q**PA**T**GA**A**S**L**.T | Q260 | W.**R**.V | R60 |
|  | T263 | L.NFSVFYY**E**.I | E180 |
|  | A266 | M.QLLRD**N**LT | N224 |
|  | L268 | L.**N**FSVFYYE.I | N173 |
