# Supplementary figures and images for: Amuc_1100 alleviates HFD-induced hepatic lipid accumulation via gut microbiota in zebrafish: insights from the role of intestinal 14-3-3β/α-A
Source: J Anim Sci Biotechnol. 2026 Jun 10;17:115. doi: 10.1186/s40104-026-01418-7 (PMC13251306; doi:10.1186/s40104-026-01418-7)

Figure 5A


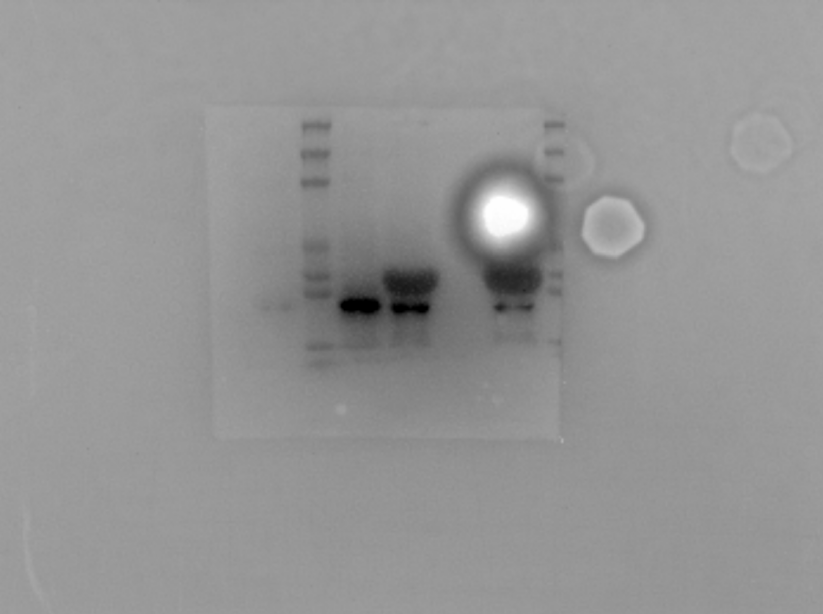


IB: Anti-HA

IP: Anti-flag

Input

25 kd

35 kd


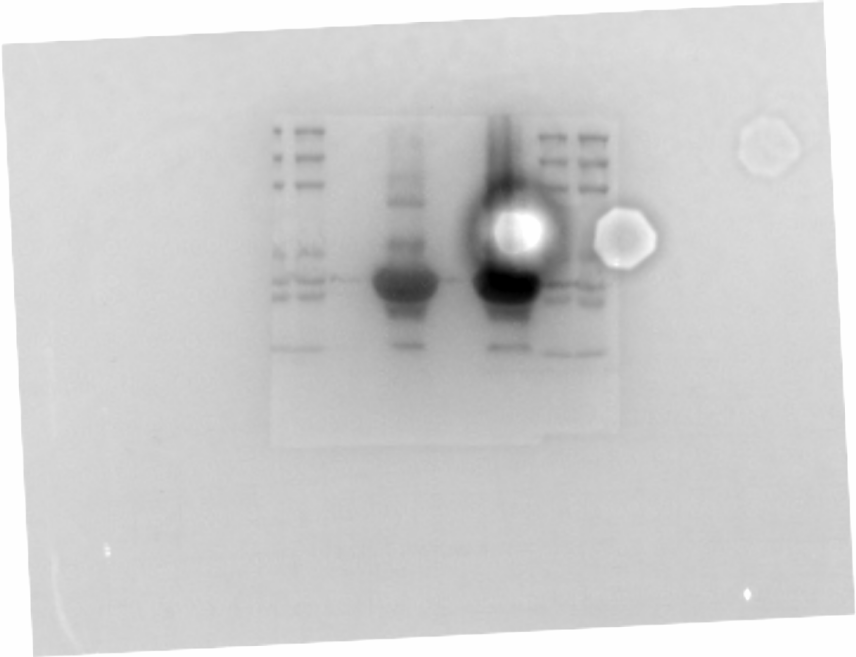


IB: Anti-flag

Input

IP: Anti-flag

40 kd

25 kd

Supplement: Supplementary file 2 — Additional file 2. Original Western blot images. [file 40104_2026_1418_MOESM2_ESM.docx]
